# Study protocol for a phase 1/2, single-centre, double-blind, double-dummy, randomized, active-controlled, age de-escalation trial to assess the safety, tolerability and immunogenicity of a measles and rubella vaccine delivered by a microneedle patch in healthy adults (18 to 40 years), measles and rubella vaccine-primed toddlers (15 to 18 months) and measles and rubella vaccine-naïve infants (9 to 10 months) in The Gambia [Measles and Rubella Vaccine Microneedle Patch Phase 1/2 Age De-escalation Trial]

**DOI:** 10.1186/s13063-022-06493-5

**Published:** 2022-09-14

**Authors:** Ikechukwu Adigweme, Edem Akpalu, Mohammed Yisa, Simon Donkor, Lamin B. Jarju, Baba Danso, Anthony Mendy, David Jeffries, Abdoulie Njie, Andrew Bruce, Michael Royals, James L. Goodson, Mark R. Prausnitz, Devin McAllister, Paul A. Rota, Sebastien Henry, Ed Clarke

**Affiliations:** 1 Vaccines and Immunity Theme, MRC Unit The Gambia at the London School of Hygiene and Tropical Medicine, Atlantic Boulevard, Fajara, PO Box 273, Banjul, The Gambia; 2 Micron Biomedical, Inc, 311 Ferst Dr, NW, Suite L1309, Atlanta, GA 30332 USA; 3 Accelerated Disease Control Branch, Global Immunization Division, Centers for Disease Control and Prevention, Atlanta, GA USA; 4 Division of Viral Diseases, National Center for Immunization and Respiratory Diseases, Centers for Disease Control and Prevention, Atlanta, GA USA

**Keywords:** Measles vaccine, Rubella vaccine, Microneedle patch, Microarray patch, Microneedle, Double dummy, Age de-escalation, Phase 1 clinical trial, Phase 2 clinical trial

## Abstract

**Background:**

New strategies to increase measles and rubella vaccine coverage, particularly in low- and middle-income countries, are needed if elimination goals are to be achieved. With this regard, measles and rubella vaccine microneedle patches (MRV-MNP), in which the vaccine is embedded in dissolving microneedles, offer several potential advantages over subcutaneous delivery. These include ease of administration, increased thermostability, an absence of sharps waste, reduced overall costs and pain-free administration. This trial will provide the first clinical trial data on MRV-MNP use and the first clinical vaccine trial of MNP technology in children and infants.

**Methods:**

This is a phase 1/2, randomized, active-controlled, double-blind, double-dummy, age de-escalation trial. Based on the defined eligibility criteria for the trial, including screening laboratory investigations, 45 adults [18–40 years] followed by 120 toddlers [15–18 months] and 120 infants [9–10 months] will be enrolled in series. To allow double-blinding, participants will receive either the MRV-MNP and a placebo (0.9% sodium chloride) subcutaneous (SC) injection or a placebo MNP and the MRV by SC injection (MRV-SC). Local and systemic adverse event data will be collected for 14 days following study product administration. Safety laboratories will be repeated on day 7 and, in the adult cohort alone, on day 14. Unsolicited adverse events including serious adverse events will be collected until the final study visit for each participant on day 180. Measles and rubella serum neutralizing antibodies will be measured at baseline, on day 42 and on day 180. Cohort progression will be dependent on review of the unblinded safety data by an independent data monitoring committee.

**Discussion:**

This trial will provide the first clinical data on the use of a MNP to deliver the MRV and the first data on the use of MNPs in a paediatric population. It will guide future product development decisions for what may be a key technology for future measles and rubella elimination.

**Trial registration:**

Pan-African Clinical Trials Registry 202008836432905. ClinicalTrials.govNCT04394689

## Administrative information

Note: the numbers in curly brackets in this protocol refer to SPIRIT checklist item numbers. The order of the items has been modified to group similar items (see http://www.equator-network.org/reporting-guidelines/spirit-2013-statement-defining-standard-protocol-items-for-clinical-trials/).

**Table:** 

Title {1}	A phase 1/2, single-centre, double-blind, double-dummy, randomized, active-controlled, age de-escalation trial to assess the safety, tolerability and immunogenicity of a measles and rubella vaccine delivered by a microneedle patch in healthy adults (18 to 40 years), measles and rubella vaccine-primed toddlers (15 to 18 months) and measles and rubella vaccine-naïve infants (9 to 10 months) in The Gambia. [Measles and Rubella Vaccine Microneedle Patch Phase 1/2 Age De-escalation Trial].
Trial registration {2a and 2b}.	Pan-African Clinical Trials Registry: 202008836432905. ClinicalTrials.gov: NCT04394689
Protocol version {3}	Version 3.1 26 July 2021
Funding {4}	Bill & Melinda Gates Foundation through a grant provided to Micron Biomedical, Inc.
Author details {5a}	Ikechukwu Adigweme^1^, Edem Akpalu^1^, Mohammed Yisa^1^, Simon Donkor^1^, Lamin B Jarju^1^, Baba Danso^1^, Anthony Mendy^1^, David Jeffries^1^, Andrew Bruce^1^, Michael Royals^2^, James L Goodson^3^, Mark R Prausnitz^2^, Devin McAllister^2^, Paul A Rota^4^, Sebastien Henry^2^, Ed Clarke^1*^. * Corresponding Author: Ed.Clarke@lshtm.ac.uk; (+220) 7039732. ^1^Vaccines and Immunity Theme, MRC Unit The Gambia at the London School of Hygiene and Tropical Medicine, Atlantic Boulevard, Fajara, PO Box 273, Banjul, The Gambia ^2^Micron Biomedical, Inc, 311 Ferst Dr, NW, Suite L1309, Atlanta, GA 30332, United States of America. ^3^Accelerated Disease Control Branch, Global Immunization Division, Centers for Disease Control and Prevention, Atlanta, Georgia, United States of America ^4^Division of Viral Diseases, National Center for Immunization and Respiratory Diseases, Centers for Disease Control and Prevention, Atlanta, Georgia, United States of America
Name and contact information for the trial sponsor {5b}	Sebastien Henry, Micron Biomedical, Inc. 311 Ferst Dr, NW, Suite L1309, Atlanta, GA 30332, USA, shenry@micronbiomedical.com
Role of sponsor {5c}	The sponsor (Micron Biomedical, Inc) worked with the MRCG investigators to develop the design of the trial and the data collection tools used and will be involved in the interpretation of the data, in report writing and in decisions to submit the data for publication. The funder (Bill & Melinda Gates Foundation) was not involved in the study design or conduct and will not be involved in data interpretation or publication.

## Introduction

### Background and rationale {6a}

#### Virology, epidemiology and immunity

#### Measles

Measles is a non-segmented, negative-sense RNA virus and a member of the *Morbillivirus* genus in the family *Paramyxoviridae.* The genome encodes six structural and two non-structural proteins. Of the structural proteins, the haemagglutinin protein is one of two transmembrane glycoproteins on the virion surface [1]. It binds to cellular receptors present on lymphocytes, monocytes, macrophages and dendritic cells as well as to receptors expressed on epithelial cells, the latter explaining the wide tissue distribution of the virus [2, 3].

Measles is most commonly spread from person to person over short distances in respiratory droplets although can also be spread by small particle aerosols which may remain suspended in the air for periods of hours [4, 5]. The median incubation period from the time of infection to the time symptoms appear has been estimated to be between 12 and 13 days with the infectious period beginning approximately 4 days before the onset of the rash and lasting for 4 days after the rash appears [6]. Estimates of the basic reproductive number (*R*_0_) for measles range between 9 and 18 and are dependent not only on the characteristics of the virus but also on the population density and social mixing. This makes measles one of the most highly transmissible human pathogens, and thus, a very high level of population immunity is required to interrupt measles virus transmission [7, 8]. However, except in cases of sub-acute sclerosing panencephalitis (SSPE), measles does not result in latent or persistent infection and has no animal reservoirs capable of independently sustaining transmission; therefore, eradication is in principal possible [1]. Except during the initial acute infection, measles is not transmittable in those with SSPE.

Immunity to measles following infection or vaccination is mediated by measles-specific neutralizing immunoglobulin G (IgG) antibodies (serum neutralizing antibodies [SNA]) directed against the haemagglutinin protein which block binding of the virus to the cell surface receptors and hence virus entry into the host cells, normally mediated by the fusion protein [9, 10]. Although 24 measles virus genotypes are recognized based on the sequence of the nucleoprotein and hemagglutinin genes, these are not associated with distinct serotypes. Instead, the virus has a single antigenic type based on conserved neutralizing epitopes of the haemagglutinin protein. For this reason, loss of vaccine efficacy associated with strain diversity or evolution has not been observed [11–13].

While antibodies are key to preventing infection, cellular immune responses to the virus are important for viral clearance and recovery [14, 15]. Following infection, a secondary immune-deficient state affecting both the innate and adaptive immune system renders individuals at an increased risk of secondary bacterial and viral infections for weeks to months or perhaps longer [16–19]. As such, secondary pneumonia is responsible for most measles-associated morbidity and mortality with complications being most common in young infants, in adults, and in those who are undernourished — particularly children with vitamin A deficiency [20].

Deaths due to measles have declined substantially over the past century — initially as a result of improvements in nutritional and other socioeconomic factors, and latterly through measles vaccination [1]. During 2000–2020, the annual number of estimated measles deaths decreased 94%, from 1,072,800 to 60,700, and an estimated 31.7 million measles deaths were averted by vaccination [21]. Case fatality ratios range from less than 1 in 1000 in high-income settings, to 5% in endemic settings in sub-Saharan Africa, and to as high as 20 to 30% in vulnerable refugee or internally displaced populations [22].

#### Rubella

Rubella is a single-stranded, positive-sense RNA virus. It is the sole member of the *Rubivirus* genus in the Togaviridae family [23, 24]. The virus has a nucleocapsid enveloped in a host-derived lipid membrane. Two glycoproteins, E1 and E2, are anchored in the external layer of the membrane [25]. The E1 protein binds to host receptors resulting in receptor-mediated endocytosis [26–28]. While the definitive host receptors have not yet been identified, E1 binds to myelin oligodendrocyte glycoprotein (MOG) which is present in the human central nervous system and gastrointestinal and placental tissue and may explain the neurological pathologies associated with congenital rubella syndrome (CRS). However, MOG is not expressed in the respiratory tract, on the skin or on lymphocytes, making it unlikely to be the cellular receptor responsible for primary rubella infections [24, 29, 30].

As with measles, rubella is spread from person to person via the respiratory route; initial rubella viral replication takes place in the buccal mucosa and the lymphoid tissue of the upper respiratory tract before the virus spreads via the lymphatic system and through the circulation of mononuclear cells [24]. The incubation period from infection to the appearance of the rash is typically around 14 days although lymphadenopathy may precede rash development. The basic reproductive number, *R*_0_, for rubella has been estimated to be between 3 and 8 in European countries although may be up to 12 in areas of high population density in low-income countries [31–33]. In urban African settings, the herd immunity threshold, required to interrupt transmission, is between 85 and 91% making elimination possible if levels of population immunity above this level can be sustained [33]. Rubella is also a candidate for worldwide eradication given that humans are the only known host and that latent or persistent infections do not occur [34, 35].

Immunity against rubella is directed against the E1 protein with neutralizing antibody titres most accurately correlating with protection from infection. However, SNA titres are less commonly measured than for measles; thus, binding IgG measured by enzyme-linked immunoassay (EIA) are also used to assess levels of rubella-specific immunity [36–39]. Furthermore, as with measles, although genotypic variation exists, these are not associated with antigenic differences in key epitopes; thus, only a single serotype consistently susceptible to vaccine-induced neutralizing antibodies has been identified [24].

In the pre-vaccination era, rubella was typically a mild self-limiting viral infection affecting children and young adults worldwide. Occasionally, the infection may be associated with more severe constitutional symptoms and high fever at the time of the illness and joint involvement ranging from transient stiffness to more significant arthritis are not uncommon but also self-limiting [34]. However, when infection occurs immediately before, or in the first trimester of pregnancy, CRS or foetal loss occurs in up to 90% of pregnancies. Congenital anomalies are rare beyond the 16th week of gestation, although sensorineural hearing loss can occur up to a gestation of 20 weeks [40]. The defects associated with CRS most commonly affect the eyes (cataracts, microphthalmia, glaucoma and chorioretinitis), ears (sensorineural hearing loss), heart (pulmonary stenosis, ventricular septal defects) and brain (microcephaly, developmental delay) [34]. Prior to rubella vaccine introduction, the incidence of CRS was reported at between 0.1 and 0.2 per 1000 live births during endemic periods and at up to 4.0 per 1000 live births during rubella epidemics [41]. In 2000, rubella-containing vaccine (RCV) had been introduced in only 13% of lower-middle-income countries and 3% of low-income countries, despite all 57 high-income countries having already introduced RCV [42]. By 2018, 39 (85%) of the 46 lower-middle-income countries and 14 (45%) of the 31 low-income countries had introduced RCV. During 2000–2018, the number of globally reported rubella cases decreased 96% from 670,894 to 26,006. By 2019, 81 countries had verified rubella elimination; however, rubella remains a leading cause of vaccine-preventable birth defects globally [42].

#### Measles and rubella vaccines

Measles vaccines were first licensed in 1963 and currently only live attenuated vaccines are available. Most measles vaccines available originate from the Edmonston strain of measles. Attenuation has been achieved through repeated passage in non-human cells. Various derived strains, including the Schwarz, Edmonston-Zagreb, AIK-C and Moraten, have less than 0.6% sequence differences in selected genes. Non-Edmonston strains show greater sequence divergence. Irrespective of these differences, measles vaccines protect equally well against all wild-type measles virus genotypes. Each 0.5-mL dose of a measles vaccine contains at least 1000 plaque forming units (PFU) of the vaccine strain virus [1, 43]. Two previously licensed vaccines, an inactivated vaccine and a high titre vaccine, have been withdrawn as a result of safety concerns [43].

Most rubella vaccines are based on the live attenuated RA 27/3 strain. The strain was first isolated from an infected foetus in the 1960s and subsequently passaged through human diploid cell lines. Other strains include the Takahashi, Matsuura and TO-336 strains, in use predominately in Japan, and the BRD-2 strain which is in use in China. Each 0.5-mL dose of a rubella vaccine contains at least 1000 PFU of the virus [34].

#### Measles and rubella vaccination

In its latest position paper on measles vaccines, the World Health Organization (WHO) recommends that reaching children with two doses of a measles-containing vaccine (MCV) should be standard for all national immunization programs [43]. In addition, it recommends that countries aiming for measles elimination should consistently achieve ≥95% two-dose coverage equitably in children across all districts [43].

In countries with ongoing measles virus transmission, the first dose of a measles-containing vaccine (MCV1) should be given at 9 months of age, following which around 85% of infants will seroconvert. Vaccination at 12 to 15 months of age increases the seroconversion rate to more than 95%, but this later age of administration is only appropriate in non-endemic settings when the risk of exposure to wild-type measles virus before this age is low [8].

In order to achieve the high levels of population immunity necessary to interrupt measles virus transmission, a routine second dose of a measles-containing vaccine (MCV2) is required at either 15 to 18 months of age or at school entry depending on local MCV1 coverage data as well as epidemiological and logistical considerations [43].

In settings with suboptimal routine vaccination coverage, periodic national or sub-national vaccination campaigns (supplementary immunization activities [SIA]) are needed. SIAs are a highly effective strategy to increase measles and rubella population immunity in settings with weak health systems and hence low Expanded Programme on Immunization (EPI) coverage. The timing and target age group for campaigns should be determined based on knowledge of coverage with routine vaccines and previous campaigns, as well as surveillance, and any available seroprevalence data. Based on mathematical models, high coverage campaigns targeting children less than 5 years of age are generally more cost effective than campaigns achieving lower coverage across a wider age range. However, since SIAs will generally be implemented using a combined measles and rubella vaccine, rubella epidemiology and rates of CRS also need to be considered in such planning decisions [44].

During outbreak response activities, and during campaigns in settings where the risk of measles before 9 months of age is high, e.g. for internally displaced populations, refugees and populations in conflict zones, for infants known to be directly exposed to a case of measles and for HIV-positive or HIV-exposed infants, the vaccine may be given as early as 6 months of age. However, a lower proportion still will seroconvert, and doses received before 9 months of age should therefore be considered as a supplementary (‘MCV0’) dose and MCV1 and MCV2 should still be given as planned.

In its latest position paper on rubella from 2020, the WHO recommends that, in light of the ongoing global burden of CRS and the effectiveness of rubella vaccination, all countries should take the opportunities offered by accelerated measles control and elimination goals to introduce rubella-containing vaccines into their routine national immunization programmes [34]. At the time of rubella vaccine introduction, it is recommended that a measles and rubella vaccine (MRV) be administered through an initial catch-up SIA targeting a wide age range, followed by prompt introduction into the routine childhood immunization programme to maintain high coverage. The timing of rubella vaccination otherwise is driven by the needs of the measles programme and thus defined by measles epidemiology and surveillance data [34].

#### Eradication and elimination of targets and barriers

Elimination is defined as the absence of endemic measles or rubella virus transmission in a defined geographic area for more than 12 months in the presence of a well-performing surveillance system. Eradication is defined as the worldwide interruption of measles or rubella virus transmission in the presence of a well-performing surveillance system [1, 24].

In 2010, the World Health Assembly (WHA) established three goals for measles control with the aim that they would be achieved by 2015 [45, 46]: first, to establish greater than 90% routine national MCV1 coverage amongst children of one year and above, including greater that 80% coverage in all districts (a level still considered insufficient to achieve elimination); second, to reduce measles incidence to fewer than 5 cases per million head of population; and third, to reduce measles mortality by 95% compared to the year 2000 figures [45, 46]. In 2012, the WHA endorsed the Global Vaccine Action Plan (GVAP), an implementation plan for the ‘Decade of Vaccines’, which set the objective of measles elimination in four WHO regions by 2015 and measles and rubella elimination in five regions by 2020. However, while all six WHO regions have now set measles elimination goals, currently, only one region has been verified as having achieved measles elimination and subsequently lost that elimination status due to re-established endemic transmission following measles virus importations [45]. The Global Measles and Rubella Strategic Plan 2012–2020 set out how countries and other partners would achieve a world without measles, rubella and CRS [35].

Between 2000 and 2016, MCV1 coverage increased from 72 to 85% globally, although with considerable regional variation: 72% in the African region (AFR), 92% in the Americas (AMR), 77% in the Eastern Mediterranean region (EMR), 93% in the European (EUR) region and 87% in the Southeast Asia region (SEAR). Only the Western Pacific region (WPR) had sustained coverage >95% since 2008 [45]. Over the same period, MCV2 coverage increased from 15 to 64%. In addition, during 2016, a further 119 million individuals received a measles vaccine during SIA conducted in 32 countries across AFR, AMR, EMR, SEAR and WPR with coverage estimates ranging from 84% to above 95% [45]. In 2016, AMR was verified as free from endemic measles while in the same year 24 EUR countries also declared elimination [45]. However, after this period of progress, a global resurgence in measles during 2017–2019 led to re-established endemic measles in countries in the Americas and Europe, as well as increased global measles incidence and morbidity and mortality. In 2019, reported measles cases had increased to 869,770, incidence increased to 120 cases per million. By the end of 2020, 81 (42%) of 194 countries had verified measles elimination [47]; however, sustained measles virus transmission occurred in all regions, and measles deaths continue to occur [21].

Thus, while great advancements toward measles elimination have been made, targets have been consistently missed, including those set for 2020 [48, 49]. In 2020, the 73rd WHA endorsed the Immunization Agenda 2030: A Global Strategy to Leave No One Behind (IA2030) [50]. IA2030 builds on the goals of GVAP and existing disease-specific initiatives and focuses on health systems strengthening to help achieve these goals, with core strategic priorities that include research and innovation. In concert, the new Measles and Rubella Strategic Framework 2021–2030 strongly emphasizes the need for investing in research and innovation for immunizations and elimination [51]. A mid-term review of the previous Measles and Rubella Strategic Plan 2012–2020 had identified a number of core strategies to drive elimination and highlighted the value of targeted, programmatically driven research aimed at removing barriers limiting programme performance [48, 49]. A survey, subsequently endorsed by the Strategic Advisory Group of Experts on Immunization (SAGE) committee, considered the development of novel ways to optimize vaccine delivery to be of high priority. The review highlighted that the most critical potentially ‘game-changing’ advance in this area was likely to be the capacity to deliver the vaccine through thermostable microneedle patches [48, 49]. The technology would allow for the delivery of vaccines by non-medically trained personnel — of critical importance in countries of limited resources. In addition, the technology potentially allows for house-to-house delivery of parenteral vaccines providing new opportunities to increase coverage [48, 49]. The Vaccine Innovation Prioritisation Strategy (VIPS) Alliance established by the Gavi Secretariat, WHO, Bill & Melinda Gates Foundation, United Nations Children’s Fund (UNICEF) and PATH, conducted a process to identify and prioritize the top three vaccine product innovations with the greatest potential to achieve vaccination coverage equity and improve immunization systems and concluded that Microarray patches (MAPs) were the highest priority as they are potentially “transformational” innovations that could overcome immunization barriers identified by low- and middle-income countries (LMICs) [52].

#### Microneedle patch technology

Microneedle patches (MNP) have the potential to overcome several of the key barriers to vaccine delivery, particularly in LMICs. MNPs are expected to be easier to administer and would reduce the need for the large number of trained and skilled clinical personnel that are currently required to deliver intramuscular (IM) or subcutaneous (SC) vaccines to large numbers of people during SIAs. MNPs with improved thermostability would facilitate the supply chain and reduce the needs for costly cold-chain maintenance procedures. MNPs also would eliminate the risks of sharps injury and hence reduce the spread of blood-borne viruses (and the consequent costs of safe sharps disposal). MNPs would save on vaccine wastage associated with the multi-dose vials used for the currently available vaccines [53].

The MNP consists of microneedles, less than 1mm in length, that deliver the vaccine into the dermis and epidermis rather than into deeper tissues. They are typically of two designs: either a solid metal or polymer-based microneedle that is coated with the vaccine and releases it when a soluble coat dissolves on application of the MNP to the skin, or a water-soluble microneedle into which the vaccine is incorporated. In the latter case, the entire microneedle dissolves on application [53]. It is this approach applied in the development of the MRV-MNP which is used in this trial.

#### Measles and rubella microneedle patches

#### Immunogenicity and safety data

An initial proof-of-concept study of measles vaccination using the MNP technology used in this study was carried out in a cotton rat model. The results showed that measles vaccine retained potency when incorporated into an MNP. When the MNP was administered to the skin, the vaccine reconstituted in situ in the skin and induced SNA titres that were comparable between the MNP and SC injection groups using similar vaccine doses [54]. A study of the measles vaccine MNP was subsequently undertaken in rhesus macaques. This again showed that measles vaccination with an MNP generated SNA titres equivalent to those generated when the same vaccine and dose was administered by the SC route. In both the cotton rats and the rhesus macaques, there was little or no reactogenicity at the site of MNP application and the vaccinations were well tolerated in all animals [55].

Following these studies, an MRV-MNP was developed, and its safety and immunogenicity were tested in rhesus macaques. This MNP vaccine served as a prototype for further patch development prior to the planned human studies. All macaques receiving either the MRV-MNP or MRV by the SC route (MRV-SC) developed concentrations in excess of the protective 120 mIU/ml for measles and 10 IU/ml for rubella by EIA. Furthermore, the antibody concentrations generated after vaccination with the MRV-MNP were equivalent to those generated following SC injection. The procedure was well tolerated and no adverse reactions to vaccination were observed in any of the macaques [56].

Finally, to simulate vaccination of human infants, 2-month-old rhesus macaques were vaccinated with the MRV-MNP [56]. The SNA responses generated by the MRV-MNP were at least equivalent to those induced when the same vaccine was injected by the SC route. All macaques vaccinated with the MRV-MNP developed titres to measles and rubella that are considered protective (>120mIU/ml for measles and >10 IU/ml for rubella) and all of the macaques vaccinated with the MRV-MNP were completely protected from challenge by a wild-type measles virus [56].

#### Patch stability

Initial studies with a measles vaccine MNP showed that an optimized formulation of a patch maintained full potency for almost 4 months at 25°C and had less than a 10-fold decrease in potency after almost 4 months at 40°C. An excipient screen was subsequently conducted to improve the stability of both measles and rubella vaccines when co-formulated into the MNP. With the new formulation, the titres of MRV were maintained for at least 6 months at 25 °C and 60% relative humidity and lost less than 0.5 log_10_ CCID50 following storage at 40 °C and 75% relative humidity for 2 weeks. Additional studies showed that the microneedles in the MRV-MNP were strong enough to penetrate and dissolve in the skin.

#### Human dissolving microneedle patch data

Placebo dissolving MNP (PLA-MNP) data as well as data on the use of dissolving MNP to deliver the inactivated influenza vaccine (IIV) in humans are available [57, 58].

#### Placebo dissolving microneedle patch — tolerability, acceptability and usability

A PLA-MNP, formulated using a comparable approach as used to formulate the MRV-MNP, has been assessed in healthy human adult subjects [58]. This MNP does not contain the active vaccine, only the water-soluble excipients designed to dissolve and release the vaccine on application.

Over a 7-day period following application, the MNP was well tolerated [58]. All participants experienced grade 1 or 2 erythema at the site of injection on the day of MNP application although this resolved in all cases by day 7 following MNP application. On day 0, 13/15 participants (87%) had grade 2 erythema and 2/15 (13%) had grade 1 erythema. On day 1, 5/15 (33%) had grade 2 erythema and 7/15 (47%) grade 1 erythema. Beyond day 1, any erythema present was of grade 1 only. One participant (7%) experienced tenderness on the day of MNP application which had resolved by the following day. No participants experienced pain or swelling at the site of MNP application. No participant experienced a grade 3 or 4 reaction of any kind. Whether administered, after brief training, by the investigator or by the participant themselves, the puncture efficiency of the microneedles into the skin was close to 100% [58]. In both cases, the microneedles dissolved as expected thus confirming the ability of minimally trained subjects to use the MNP.

#### Inactivated influenza vaccine dissolving microneedle patches — safety, immunogenicity and acceptability

A dissolvable MNP for the administration of the trivalent inactivated influenza vaccine (IIV) formulated using a comparable approach to that used for the MRV-MNP has been assessed in a randomized, partly blinded, placebo-controlled trial in healthy adult participants [57].

Four groups of 25 participants were compared: IIV administered by IM injection (IM-IIV), IIV administered using an MNP by a healthcare worker (MNP-IIV-HCW), IIV administered using an MNP by the participant (MNP-IIV-self) and PLA-MNP.

#### MNP-IIV safety

No treatment-related serious adverse reactions were reported, and no participants withdrew due to an adverse reaction [57].

Local adverse events (AE) observed in the MNP-IIV-HCW and the MNP-IIV-self groups were similar and mostly mild. The IM-IIV group had a higher incidence of grade 2 and 3 AE (three (12%) of 25 participants) than either of the MNP-IIV groups (one [2%] of 50 participants in the MNP-IIV groups combined; *p* = 0.02). Significantly more local AE were reported in the MNP-IIV groups than in the IM-IIV group: pruritus (41 [82%] of 50 participants versus four [16%] of 25 participants; *p* < 0.0001) and erythema (20 [40%] of 50 versus zero of 25; *p* = 0.0002). The most common vaccination site reaction for the two MNP-IIV groups was pruritus; 36 (88%) of these reactions in 41 participants were mild and self-limiting, lasting 2 to 3 days on average. In the IM-IIV group, injection site pain reported over the days after vaccination was more than twice as frequent (11 [44%] of 25 participants versus ten (20%) of 50; *p* = 0.05) and more severe (grade 2 or higher; three [12%] of 25 versus one [2%] of 50; *p* = 0.1) compared with the MNP-IIV groups combined [57].

The rate and severity of solicited systemic AE did not differ among the groups receiving the IIV. Among vaccinated groups (MNP-IIV-HCW, IM-IIV and MNP-IIV-self), the overall incidence of solicited adverse events (*n* = 89 versus *n* = 73 versus *n* = 73 respectively) and unsolicited AE (*n* = 18 versus *n* = 12 versus *n* = 14 respectively) was similar [57].

No new chronic medical illnesses or influenza-like illnesses were reported. Sixty-one unsolicited AE were reported by 41 (41%) of 100 participants after receiving the assigned treatment. Few treatment-unrelated grade 3 events were reported. One participant in the MNP-IIV-self group developed acute enteritis requiring hospital treatment, and another participant in the PLA-MNP group developed grade 3 hypertension while off her hypertensive drugs. One participant in the MNP-IIV-self group had rhabdomyolysis due to strenuous exercise at baseline before receipt of the study product, and another participant in the IM-IIV group had a grade 3 elevation in liver function test due to exercise and excessive alcohol and paracetamol consumption 30 days after vaccination. These laboratory abnormalities resolved spontaneously. There were 13 treatment-related AE (seven in the PLA-MNP group, three in the IM-IIV group and three in the MNP-IIV-HCW group) reported in eight participants. These AEs were mostly grade 1 laboratory events (thrombocytopenia, leukopenia and neutropenia), all of which resolved during study follow-up. No grade 3 or higher treatment-related laboratory AE occurred [57].

#### MNP-IIV immunogenicity

The geometric mean titres (GMT) determined by haemagglutination inhibition (HAI) antibody assay were similar at day 28 between the MNP-IIV-HCW group and the IM-IIV group for all influenza virus strains: H1N1 strain (1197 [95% CI 855–1675] versus 997 [95% CI 703–1415]), H3N2 strain (287 [95% CI 192–430] versus 223 [95% CI 160–312]) and B strain (126 [95% CI 86–184] versus 94 [95% CI 73–122]). Similar GMT were seen in the MNP-IIV-self group [57].

When comparing immune responses in the MNP-IIV-HCW and the IM-IIV groups, seroprotection and seroconversion rates at day 28 were similar for all three influenza strains contained in IIV and were significantly higher than the equivalent rates in the placebo group (all *p* < 0.01). The only exception was the day 28 H3N2 seroprotection rates which were similar between groups. There was a higher seroconversion percentage against the B strain for the MNP-IIV-HCW and MNP-IIV-self groups combined (31 [65%] of 48 participants [95% CI 60–78]) compared with the IM-IIV group (eight [32%] of 25 [95% CI 15–54]; *p* = 0.01). Seroprotection against the three influenza strains 6 months after vaccination was seen in 20 to 24 (83–100%) of 24 participants in the MNP-IIV-HCW group and in 20 to 25 (80–100%) of 25 participants in the IM-IIV group. The MNP-IIV-self group had similar seroprotection, with 18 to 24 (75–100%) of 24 participants having an HAI titre of 1:40 or higher at 180 days later [57].

#### MNP-IIV usability and stability

Intramuscular vaccination delivered at least 15μg of each influenza antigen. Measurement of residual antigens in the 50 MNP-IIV patches used in the study showed that the mean dose delivered by MNP-IIV was 11.3μg for the H1N1 strain, 14.4μg for the H3N2 strain and 13.1μg for the B strain. No significant difference was reported between the dose of each strain delivered by the MNP-IIV-HCW and MNP-IIV-self groups, suggesting that the participants were able to correctly self-administer MNPs. After vaccination, imaging of used MNPs showed that the microneedles had dissolved in the skin, suggesting that the used patches could be discarded as non-sharps waste. After storage in desiccated packaging at 5°C, 25°C and 40°C for 12 months, IIV potency for all three strains in the MNP-IIV remained within product specifications which supports the storage of patches without refrigeration.

#### MNP-IIV acceptability

Immediately after vaccination, 48 (96%) of 50 participants who received MNP-IIV reported no pain during MNP application, but only 18 (82%) of 22 participants reported that IM injection was painless (*p*=0.04). On a scale of 1 (negative experience) to 5 (positive experience), participants in the MNP groups reported high acceptability for MNP vaccination, with mean scores between 4.5 and 4.8 across the IIV and placebo groups. Participants receiving IM-IIV reported a mean score of 4.4 which was not significantly different from the MNP groups (*p*=0.07). When asked on day 28 (thereby assessing the complete vaccination and post-vaccination experience), 33 (70%) of 47 MNP-IIV recipients preferred MNP vaccination over other vaccination routes as a delivery method for future influenza vaccination (*p* < 0.0001) suggesting a positive experience with MNP patch vaccination. Five stated that they preferred no vaccine [57].

### Trial rationale and relevance to The Gambia

This phase 1/2, single-centre, double-blind, double-dummy, randomized, active-controlled, age de-escalation trial will provide the key safety and immunogenicity data on which to make future critical decisions regarding MRV-MNP product development.

The future availability of thermostable MNP for the delivery of the MRV has been described as potentially ‘game changing’ to the global measles and rubella eradication strategy, given the expectation that such technology will facilitate an increase in vaccination coverage — both through routine immunization services and through SIAs [48, 49]. Such impact is expected to be most keenly felt in the African region which to date has achieved the lowest MRV coverage of any WHO region [48, 49, 59]. This reflects, in part, the limited availability of skilled healthcare personnel able to deliver parenteral injections in the region, particularly during SIAs. Also, to constraints related to maintenance of the cold-chain and the disposal of sharps waste. As a result, almost two-thirds of measles deaths worldwide occur in the African region.

Despite The Gambia having a generally efficient EPI programme, the level of measles vaccine coverage remains considerably below that required to prevent outbreaks without regular national SIAs [59]. Measles and more recently measles and rubella SIAs have consequently been required every 3 to 4 years since 2003 (2003, 2007, 2011, 2013, 2016, due 2020 but now delayed). Based on the most recent Gambian Bureau of Statistics/UNICEF Multiple Indicator Cluster Survey (MICS), published in July 2019, the coverage with a first dose of the MRV by 12 months of age in The Gambia is 82.4%. This rises to 86.8% when children up to 23 months of age are included. The coverage with the recommended second dose of the vaccine at 24 months of age is 64.1%. This only rises to 67.1% including children up to 35 months of age. Therefore, even assuming all children were protected by a single dose of the vaccine, the vaccine-induced immunity in children is considerably below minimum 92% level required to prevent measles outbreaks. Our own published data indicate that only around 80% of Gambian infants will be protected by a single dose of the vaccine [60], suggesting that the 86.8% coverage provided a population immunity level of only around 70% in those cohorts. Reflecting this, the last significant measles outbreak occurred in The Gambia in the first part of 2016. More than 120 cases were officially confirmed. This outbreak was controlled by a national MRV SIA conducted in April of the same year. Further sporadic cases of measles continue to be reported. Indeed, there were at least 12 laboratory-confirmed cases of measles in the Western region of The Gambia in the first quarter of 2020 — reflecting the accumulation of measles-susceptible children since the last campaign in 2016. Some of these cases were in children from neighbouring countries with lower vaccination coverage. This reinforces the critical need to maintain high levels of immunity in the Gambian population if significant outbreaks are to be avoided. The first-dose measles coverage in Senegal is even lower than in The Gambia, being 65.0% and 70.5% at 12 and 24 months of age respectively [61]. Coverage in Guinea-Bissau is similarly low. The additional risk of measles being imported is therefore significant given the movement of the population in the sub-region.

The same concerns apply to rubella vaccination coverage. In this case, failure to generate adequate immunity in childhood risks a population of women entering their period of childbearing without rubella immunity and being exposed during pregnancy through ongoing low-level virus circulation. Again, our own published data suggest 10% of infants will have been exposed to rubella by 9 months of age. This is supported by ongoing case detection with close to 150 laboratory-confirmed cases being reported before the national SIA in 2016. This is a concern when vaccine-induced immunity is partial, as likely to be the case in The Gambia, meaning that many women are no longer likely to be immune through natural infection in childhood, but that virus circulation is not sufficiently suppressed to reliably prevent women becoming exposed when pregnant. In this case, the risk of CRS can increase despite the vaccine programme [60, 62].

Consequently, The Gambia remains dependent on MRV SIAs, and novel approaches to facilitate campaigns and increase routine coverage while also minimizing the burden on the health sector are highly relevant. The MRV-MNP offer important potential advantages over conventional Needle and Syringe (N&S) administration in both situations.

For both routine and campaign-based delivery, the absence of sharps is a significant, but perhaps under-recognized, benefit of the MNP. A recent meta-analysis looking at sharps’ injuries in health care personnel in West Africa reported an annual exposure to a percutaneous injury (an injury with a potentially infected sharp penetrating the skin) of 36.0% and a life-time exposure of 52.9% [63]. Similar figures were reported across other African sub-regions and elsewhere [63, 64]. This means that, every year, over one-third of front-line health care professionals in this setting are potentially exposed to blood-borne infections by this route. The risk of needle stick injuries is likely to be amongst the highest in a public health officer delivering EPI vaccines given the very high throughput of infants and children and the numerous injections administered. These can be readily appreciated witnessing any routine EPI clinic at major health centres in The Gambia. The risks are likely to be higher still during community-based vaccination campaigns — particularly when involving less experienced staff. Post-exposure prophylaxis for HIV may not always be available, even assuming injuries are consistently reported. Hepatitis B vaccines do not protect all individuals — even when a full vaccine course has been received, and hepatitis C and other blood-borne infections are further risks in The Gambia as elsewhere. As such, the availability of the MRV in a form which removes this risk represents a significant benefit of direct relevance in The Gambia. In addition, the safe storage and disposal of sharps waste is associated with an additional burden on the local health system.

The increase in thermostability of the MRV in the MNP compared to the lyophilized presentation offers additional important advantages in The Gambia. Gavi, the Vaccine Alliance, estimated the cold-chain requirements in low-income countries increased fourfold over the 10 years to 2020. While this has cost implications, it also adds significantly to the logistical challenges of ensuring vaccines are delivered in good condition — particularly during campaigns when vaccines are needed across numerous community locations. The future WHO-approved use of the MNP outside the traditional ‘2–8°C’ cold-chain requirements has the potential to markedly reduce this logistical challenge compared to the standard lyophilized preparation, as well as to reduce the associated costs of cold-chain maintenance.

In The Gambia, the ease of administration is a particular benefit when considering the use of the MNP for SIA-based delivery. The MRV campaign in 2016 targeted 802,000 Gambians between 9 months and 14 years of age. The availability of sufficient clinical personnel to deliver this number of SC injections stretches resources and adversely affects other services both running up to and during the campaign period. This contrasts with oral polio vaccine campaigns that utilize large numbers of volunteers from youth and other community advocacy groups to deliver the vaccines. The MNP are similarly designed to be reliably administered by non-clinical personnel following a brief pictorial explanation, thus reducing the burden placed on limited health resources and health personnel by future campaigns.

The cost of delivering measles vaccines by MNP has been estimated to be less than 60% of the costs of delivering the same vaccine SC [65]. This accounts for factors including cold-chain maintenance, personnel, supplies, transport and vaccine wastage. It does not account for the cost associated with needle stick injuries in terms of health care and lost earning which represent additional savings associated with the patches. The cost of the last MRV SIA in The Gambia was US$ 1,323,807 and this is only likely to increase in future SIAs. While much of this was met by donor support, this is still money that is not available for other activities and even the US$ 83,767 committed by The Government of The Gambia is significant.

As described above, MNP administration is essentially pain free. Pain associated with the injection is only one of many factors associated with non-attendance for routine immunizations. Data from The Gambia, undertaken with the government EPI team, examined the attitudes, perceptions and practices of the parents of infants in The Gambia related to vaccine injections [66]. Among parents, 35.7% reported that they were not comfortable with their infant receiving more than two injections at a single visit. As more vaccines are introduced into the schedule, such understandable concerns become more problematic as a barrier to vaccination.

A key element to ensure that two-dose MRV coverage attains the levels required for elimination is to ensure that opportunities for vaccination are not missed. However, MRV vaccines are supplied in 10 and 20 dose vials and vaccine wastage is a concern. Therefore, infants and children who are due or overdue this vaccine may be asked to return to future clinics if insufficient numbers are present on a given day. In many parts of The Gambia, outreach clinics, for example to smaller communities, are only conducted monthly. On this basis, MRV will be further delayed, even assuming a parent reattends. The MNP are provided in single-dose packets therefore can be provided to single infants and children without delay or wastage. Consequently, they are expected to facilitate opportunistic vaccination to a degree which is not possible when multidose vials are used.

Generating early data on the MRV-MNP in this setting is appropriate and will minimize the timeline for MRV-MNP development. Serological data from a population of adults recruited based on comparable inclusion and exclusion criteria to those to be used in this trial suggest that the baseline measles-specific SNA titres and rubella-specific IgG concentrations are likely to be high. This is expected to limit the insights regarding the immunogenicity of the vaccine in this group. For this reason, age de-escalation to the future target toddler and infant age groups, based on the safety data from the preceding cohort, is seen as fundamental to gaining the information required on both the safety and immunogenicity of MRV-MNP to base key product development decisions. Fifteen to 18-month-old toddlers are the target age group for MCV2, while 9-month-old infants are the target age group for MCV1 based on current WHO recommendations [43].

The double-dummy design, which means that all participants receive an MNP and a SC injection, is used for several reasons. First, the design ensures that the safety data the trial generates are as robust as possible. Neither the participant/parent, nor the person administering the study product, nor the staff collecting safety or immunogenicity endpoint data will be aware of the group to which a participant belongs. Thus, conscious or sub-conscious reporting bias by participants/parents and observer bias by trial staff are prevented. Second, all participants receive an MRV whether administered by MNP or SC. In both the toddler and infant groups, MRV is due within the EPI schedule in The Gambia at the age specified. The double-dummy design ensures MRV is not delayed in either group. Third, the design is efficient within the defined sample size and avoids the need for the separate PLA-MNP and MRV-SC groups which would otherwise be required. Fourth, it is likely, given the ‘novelty’ of the patches that most participants/parents will wish to experience the use of the MNP. The double-dummy design means that all participants will experience the application of the MNP and will also be able to provide feedback on their experience of its use. Finally, based on information collected to date, MNP application is not painful. Therefore, participants are not subjected to two painful procedures through the double-dummy design. Rather, all participants have a single SC injection as well as experiencing the MNP application.

The trial will provide descriptive data aiming to determine that the MRV-MNP:

- Is safe (i.e. that it has a safety and tolerability profile that is comparable to that generated following the administration of the same vaccine by the SC route)
- Is immunogenic (i.e. that it induces measles and rubella-specific immune responses that are comparable in magnitude and duration to the response generated by the SC-administered MRV)

As an early phase trial with an appropriately limited sample size, no hypothesis testing is planned.

#### Potential risks and benefits

#### Known potential risks

The safety profile of the MRV when administered by the SC route is well established. The vaccine in the MRV-MNP is the same vaccine, manufactured by the Serum Institute of India Pvt Ltd, that children in The Gambia and in many other countries across the world already receive. Local reactions at the site of injection are common and mild fever and/or rash occur in a small proportion of participants following the measles component of the vaccine but are self-limiting. Transient arthralgia or arthritis is not uncommon in rubella-naïve adolescent and adult females following the rubella component of the vaccine. Low-grade fever and rash are also common following rubella vaccination and are self-limiting. More serious reactions following the SC-administered vaccine are rare.

Human data on both a PLA-MNP and on the IIV-MNP, as well as data from animal models including non-human primates on measles vaccine MNP and MRV-MNP, support the safety of the alternative administration method. The patches themselves are composed of pharmacologically inactive excipients listed in the US Food and Drug Administration’s (FDA’s) Inactive Ingredient Database for Approved Drug Products and are already used in parenteral medications. As such, they are not in themselves expected to significantly alter the safety profile of the MRV. Local AE following MNP-based vaccine administration tends to be more frequent than following N&S-based administration but is mild and self-limiting. No significant differences in the occurrence of solicited systemic AE or unsolicited safety events were reported following IIV-MNP compared to either placebo MNP or IIV delivered by the IM route.

Serious and/or severe reactions can occur following any vaccine and can be life-threatening. This trial will provide the first human data on the administration of the MRV using an MNP. While the safety data on both the vaccine itself and the MNP-based delivery method are supportive, the possibility of unpredicted reactions remains. To minimize any risk, the eligibility and screening procedures will ensure that only healthy participants are recruited. In addition, a sentinel dosing cohort will be used at the start of the trial to minimize the number of participants exposed to the intervention on a single day. Safety data including safety laboratory evaluations will be assessed in the sentinel group prior to further recruitment proceeding. In the toddler and infant cohorts, recruitment will also start slowly, with only a small number of participants being recruited each day over the first week.

Delaying the administration of the other EPI vaccines due until the day 42 visit is not associated with any substantial risk given the risks of the diseases covered by these vaccines in the indicated window is low and/or children will already have been vaccinated against these conditions (yellow fever, poliovirus type 1 and 3 and meningococcus group A, diphtheria, tetanus and pertussis).

#### Known potential benefits

Screening procedures may identify undiagnosed health problems allowing for referral to an appropriate healthcare facility based on good medical practice in The Gambia. Unrelated conditions diagnosed during trial enrolment will be referred in the same way. At the end of their participation in the trial, all toddler and infant participants will be offered an additional SC dose of a MRV with the aim of ensuring no participant remains unprotected from either measles or rubella at the end of the trial. Such administration will be undertaken outside the trial protocol. Adults are likely to benefit from additional vaccine-induced protection against measles and rubella, as they would not otherwise be eligible for the vaccine. The MRV-MNP has the potential to have a profound impact on efforts to achieve measles and rubella elimination. Measles is a serious illness which continues to be associated with high rates of mortality in sub-Saharan Africa while CRS results in both pregnancy loss and birth defects causing significant long-term disability. Participants may therefore benefit in the knowledge that they have played their part in reducing the incidence of, and ultimately eliminating these conditions in the future.

### Objectives {7}

#### Primary — safety and tolerability

- To assess the safety and tolerability of a single dose of a measles and rubella vaccine administered by a microneedle patch (MRV-MNP) compared to a single dose of a measles and rubella vaccine administered subcutaneously (MRV-SC) in healthy adults (18-40 years), measles and rubella vaccine (MRV)-primed toddlers (15–18 months) and MRV-naïve infants (9–10 months) in The Gambia through to 180 days following study product administration.

#### Secondary — immunogenicity

- To evaluate the immunogenicity of a single dose of MRV-MNP compared to a single dose MRV-SC in healthy adults (18–40 years), MRV-primed toddlers (15–18 months) and MRV-naïve infants (9–10 months) in The Gambia through to 180 days following study product administration

#### Exploratory objectives

- To enumerate and characterize the measles and rubella-specific T-cell responses generated by MRV-MNP and MRV-SC
- To describe the experience of participants and parents following study product administration by MNP compared to SC injection
- To describe the views of trial personnel regarding study product administration by MNP and to SC injection

Exploratory objectives will be assessed in a subset of participants and will not be reported as part of the primary trial analysis.

### Trial design {8}

This is a phase 1/2, single-centre, double-blind, double-dummy, randomized, active-controlled, age de-escalation trial.

All participants will receive either the MRV-MNP and a placebo (0.9% sodium chloride) SC injection (PLA-SC) or a placebo-microneedle patch (PLA-MNP) and MRV by the SC route (MRV-SC). Only those study staff randomizing participants and preparing the study products for administration will be aware of the group to which a participant belongs. Those administering the study products, all other trial staff collecting safety and immunogenicity endpoints, and the participants and parents will be blind.

Decision regarding age de-escalation will be based on a review of the safety data from the preceding cohort (adults for toddlers and toddlers for infants) up to day 14 post-study product administration by an independent data monitoring committee (DMC).

In all, 45 adults (18 to 40 years of age) will be randomized in a 2:1 ratio. Thus, 30 adults will receive MRV-MNP and PLA-SC and 15 adults will receive MRV-SC and PLA-MNP. A total of 120 toddlers (15 to 18 months of age) will be randomized in a 1:1 ratio. Thus, 60 toddlers will receive MRV-MNP and PLA-SC while the same number of toddlers will receive MRV-SC and PLA-MNP. A total of 120 infants (9 to 10 months) will also be randomized in a 1:1 ratio. Thus, 60 infants will receive MRV-MNP and PLA-SC while the same number of infants will receive MRV-SC and PLA-MNP.

Solicited local and systemic adverse events (AEs) will be collected daily from all participants from the day of study product administration to day 13 post-study product administration. Unsolicited AE and serious adverse events (SAEs) will be collected from the day of study product administration to day 180 post-study product administration. All participants will have laboratory investigations (human immunodeficiency virus [HIV], hepatitis B, hepatitis C, haematology and biochemistry) conducted as part of screening. Adults will have safety laboratory investigations repeated on day 7 and day 14 post-study product administration. Toddlers and infants will have safety laboratory investigations repeated on day 7 post-study product administration only. All cohorts could have further laboratory investigations undertaken as clinically indicated.

All participants will have measles and rubella-specific serum neutralizing antibody (SNA) titres and measles and rubella-specific immunoglobulin G (IgG) concentrations measured at baseline and on day 42 and day 180 post-study product administration.

Statistical analysis will be descriptive and aims to generate the safety and immunogenicity data required for future go/no-go decisions related to future MRV-MNP development.

## Methods: participants, interventions and outcomes

### Study setting {9}

The study will be conducted by MRC Unit The Gambia at the London School of Hygiene and Tropical Medicine (MRCG), with recruitment and all participant-related trial procedures being undertaken within specifically designed clinical trial facilities within a government hospital (Bundung Maternal and Child Health Hospital) in the West Coast region of The Gambia, West Africa. The clinical trial facilities are a short drive from the main laboratory, vaccine storage, biobanking, data management and administrative facilities of MRCG. The MRCG clinical services department (CSD), where any inpatient medical care required by study participants would usually take place is also on the same site and thus is readily accessible.

### Eligibility criteria {10}

Participants must meet all the inclusion criteria and none of the exclusion criteria to be eligible to participate. No screening procedures will take place before an individual has provided written informed consent to join the trial.

#### Inclusion criteria

A prospective participant must meet all the following inclusion criteria to be eligible for enrolment (randomization and vaccination):

Participants must:

- Provide voluntary written/thumb-printed informed consent for trial participation (adult cohort)
- Have voluntary written/thumb-printed informed consent provided for them by a parent (toddler and infant cohort)
- Be between 18 and 40 years inclusive on the day of consent. They will be eligible from the day they reach 18 years of age until the day before they reach 41 years of age (adult cohort)
- Be between 15 and 18 months of age inclusive on the day of consent. They will be eligible from the day they reach 15 months of age until the day before they reach 19 months of age (toddler cohort)
- Be between 9 and 10 months of age inclusive on the day of consent. They will be eligible from the day they reach 9 months of age until the day before they reach 11 months of age (infant cohort)
  The identity and age of all prospective participants must be as confirmed from a suitable source document prior to informed consent. Suitable source documents include but are not limited to the birth certificate, national identification card, passport and, in toddlers and infants, the parent-held infant welfare card (IWC). Photographic identification is not consistently available and is not required. Participants/parents will be issued with a trial photographic identification card once randomization and vaccination have taken place (visit 1).
- Be judged to be able to comprehend and comply with study requirements and procedures and must be willing and able to return for all scheduled follow-up visits (adult cohort)
- Have a parent who is judged to be able to comprehend and comply with study requirement and procedures and is willing and able to return for all scheduled follow-up visits (toddler and infant cohort)
- Be willing to avoid consumption (ingestion and topical application) of herbal or other local traditional medications throughout the course of the study. Also, be willing to avoid the use of medications (for example those available for purchase at local pharmacies) except those provided by the trial team (unless in an emergency) (adult cohort)
- Have a parent who is willing to ensure they avoid consumption (ingestion and topical application) of herbal or other local traditional medications throughout the course of the study. Also, who is willing to ensure they avoid the use of medications (for example those available for purchase at local pharmacies) except those provided by the trial team (unless in an emergency) (toddler and infant cohort)
- Have a readily identifiable place of residence within a reasonable travelling distance of the clinical trial site
  This aims to ensure home visits for solicited AE can be undertaken reliably and that the participant is able to present to the trial site or be reviewed at their home in the event of other unsolicited health complaints. No specific geographical limits are set with this regard. Rather, such decisions will be made based on the judgement of senior members of the field team based on their detailed knowledge of local geography and transport links.
- Have a consistent means of telephone contact for the duration of trial participation (adult cohort)
- Have a parent with a consistent means of telephone contact for the duration of trial participation (toddler and infant cohort)
  A telephone on a closed user group (CUG) network with the field team will be provided to participants/parents to ensure they are able to contact the investigator team at any time day or night without the need for telephone credit.
- Have a site on one wrist that is judged to be suitable for MNP administration
- Adult female cohort only: have a negative serum pregnancy test at screening (visit 0) and negative urine pregnancy test on the day of vaccination (visit 1)
- Adult female cohort only: employ an effective method of birth control for 2 months preceding and throughout the study
  Effective methods of birth control are defined as follows: credible history of continuous abstinence from heterosexual activity as a normal lifestyle choice, hormonal contraceptives (oral, injectable, implant, patch and ring), barrier contraceptives (condom or diaphragm, with spermicide) and intrauterine device. When using contraceptives, participants must have been using their current contraceptive for the past 2 months to be eligible. Adult female participants with documented sterilization via tubal ligation or hysterectomy may be enrolled although for completeness all female participants will undergo pregnancy testing as outlined above. Participants will never be encouraged to start using contraception to allow them to be eligible to join the study.
- Toddler cohort only: have been parenterally vaccinated against measles and rubella at between 9 and 12 months of age
- Be willing to avoid MRV administration for the duration of enrolment in the study, including in the case of a national MRV campaign in The Gambia (adult cohort)
- Have a parent who is willing to ensure they avoid MRV administration for the duration of enrolment in the study, including in the case of a national MRV campaign in The Gambia (toddler and infant cohort)
  All toddlers and infants in the study will receive an additional SC dose of an MRV so will not miss out. Adults are not routinely included in MRV campaigns but will be given any missed doses under these circumstances.
- Be willing to avoid all vaccines not given by the trial team for the duration of the study except for non-measles and rubella vaccines given in national campaigns
- Have a parent who is willing to ensure they avoid all vaccines not given by the trial team for the duration of the study except for non-measles and rubella vaccines given in national campaigns (toddler and infant cohort).

#### Exclusion criteria

A prospective participant will not be eligible for enrolment if they meet any of the following exclusion criteria.

Participants must not:

- Have used any investigational product within the 90 days prior to study product administration or plan to use any investigational products during the period of study participation
- Have consumed (by ingestion or topical application) any herbal or other traditional medication within 14 days of study product administration
- Have a history of serious reactions to any prior vaccination or known hypersensitivity to any component of the MRV-MNP, MRV-SC, or PLA-MNP including polyethylene foam with acrylic adhesive, silicone-coated Kraft paper, stainless steel and severe allergic reactions to cow’s milk
- Have a history of anaphylactic shock or other life-threatening allergic reactions
- Have any chronic, clinically significant pulmonary, cardiovascular, hepatobiliary, gastrointestinal, renal, neurological or haematological abnormality or illness that requires medical therapy, as determined by medical history, physical examination and laboratory assessment
- Have a history of administration of any non-study vaccines within the 56 days before the administration of study products or planned vaccination during study participation, except for non-measles and rubella catch-up/national campaign administered through the Gambian Ministry of Health
- Have a history of chronic administration (defined as more than 14 consecutive days) of immunosuppressant (> 0.5mg/kg/day of prednisolone or equivalent) or other immune modifying drugs within the 12 months prior to the administration of the study vaccine including the use of glucocorticoids. The use of inhaled/per nasal glucocorticoids will be permitted. The use of topical glucocorticoids within 12 months is not permitted (specific enquiry regarding the use of skin lightening creams should be made)
- Have a history of the administration of immunoglobulins and/or any blood products within the 12 months prior to administration of the study vaccine or anticipation of such administration during the study period
- Have a history of known disturbance of coagulation or blood disorder that could cause anaemia or excess bleeding (e.g. sickle cell disorders, thalassemia and coagulation factor deficiencies)
- Have a history of keloid formation
- Have significant scars, tattoos, rashes or other dermatologic conditions in the area of the vaccination site which will interfere with the application of the MNP and assessment of local solicited AE
- Have HIV, hepatitis B or hepatitis C infection based on screening laboratory investigations
- Have any medical or social condition that in the opinion of the study clinician may interfere with the study objectives, pose a risk to the participant or prevent the participant from completing the study follow-up
- Be an employee of or direct descendant (child or grandchild) of any person employed by the investigator or sponsor
- Have plans to travel outside the study area for an extended duration during the period of study participation
  This is particularly critical in the first 42 days following study product administration during which any travel will be actively discouraged. At later timepoints, short trips within The Gambia can generally be accommodated
- Have any screening laboratory test with a toxicity score of ≥ 2 or with a toxicity score of 1 which is nonetheless judged to be clinically significant by the trial clinician. If judged to be clinically indicated, each laboratory assessment may be repeated once during the screening period, with the most recent laboratory value being used for evaluation of exclusion criteria. However, abnormal laboratory investigations will not ‘routinely’ be repeated unless there is a clinical indication as to why the initial result was abnormal and it is considered likely that the abnormality will have resolved
- Have any vital sign (heart rate, respiratory rate, non-invasive blood pressure [BP] [adult cohort only]) with a toxicity score of > 1. An abnormal vital sign may be repeated once during the screening period for a participant to remain eligible for randomization with the most recent set of vital signs being used to determine final eligibility§
- Have an axillary temperature of > 37.5°C and have had a documented fever at the same level in the 72 h preceding randomization and vaccination§
- Have a history of an illness with a fever and rash suggestive of measles in the preceding 2 months.
- Have any acute illness (severity grade > 2)§
- Have a positive rapid diagnostic test (RDT) (or blood film) for malaria. If a participant initially has a positive RDT and is then treated, a blood film will be undertaken if the RDT remains positive, to confirm treatment success given an RDT may remain positive even following successful treatment§
- Adult cohort only: have been vaccinated against measles or rubella in the preceding 4 years
- Adult cohort only: have a body mass index (BMI) of < 18.5kg/m^2^ (underweight) or > 35kg/m^2^ (severely obese)
- Adult cohort only: have a recent history (within the past year) or signs of alcohol or substance abuse
- Adult cohort only: have a history of major psychiatric disorder
- Adult cohort only: have a history of blood donation within three months of study enrolment or plans to donate blood during participation in the study
- Adult female cohort only: be pregnant or breast-feeding
- Toddler and infant cohort only: have been vertically exposed to HIV based on maternal history (mothers of potential participants will not be tested for HIV as part of screening)
- Toddler and infant cohorts only: have a weight for height *z*-score below −2SD (moderate malnutrition)
- Infant cohort only: have been vaccinated against measles or rubella
  §Participants with an acute illness, fever, other abnormal vital signs or a positive malaria RDT test may return once for a repeat screening visit within the 2-week screening period and still qualify for randomization if the acute illness has resolved. A minimum of 72 h following a documented fever (axillary temperature ≥ 37.5°C) must pass before a participant can be re-screened and vaccinated. In general, illnesses lasting more than the 2-week screening window will be considered to define a potential participant as a screen failure although a participant providing ongoing informed consent could be fully rescreened under such circumstances in the absence of any other reasons to define them as such.
  Specific exclusion criteria (vital signs, clinical examination, history of acute illness, blood test for malaria, urinary pregnancy test [adult female cohort only]) will be reassessed at the time of visit 1 and prior to confirming final eligibility and proceeding to randomization to ensure only those participants appropriate for vaccination on the day are included in the study.

All eligibility criteria will be assessed by a study clinician delegated by the principal investigator who will have overall responsibility for participant eligibility. The laboratory assessments undertaken as part of eligibility screening will be undertaken in the MRCG ISO15189-accredited CSD laboratories.

### Who will take informed consent? {26a}

#### Informed consent procedures

Informed consent for the trial will be obtained by study field workers and nurses with appropriate English and local language skills. However, in addition, all participants/parents will speak to the study doctor before final agreement to ensure any outstanding questions, particularly anything of a medical nature have been fully and accurately addressed.

Consent can only be obtained from adults who are at least 18 years old. Therefore, if both parents of a toddler or infant are under 18 years of age, the child cannot be enrolled in the study.

Informed consent is the process of ensuring that potential study participants/the parents of potential study participants understand the purpose of the study, what participation will involve, the potential risks and benefits of participation, their rights and commitments as study participants and all other information set out in the informed consent document (ICD) (as per ICH-GCP E6 (R2) section 4.8.10). Prior to any study-specific procedures taking place, signed/thumb-printed, timed and dated, written informed consent for trial participation must be obtained using an ICD approved by the Gambia Government/MRC Joint Ethics Committee and the LSHTM Research Ethics Committee. A certified copy of the completed ICD will be provided to the participant/parent at the end of this process. The outcome of the informed consent process (i.e. consented/did not consent) will also be captured in the consent log for the trial.

The entire informed consent process may be undertaken either in English or in one of several local languages for participants who are not sufficiently English literate.

Written informed consent will only routinely be obtained once at the start of the trial irrespective of the interval between consent and screening (or re-screening if applicable). However, a participant/parent will be given the chance to ask any questions at each study visit and ongoing willingness to participate will be confirmed and documented. If new information becomes available during the course of the trial which may be relevant to a participant’s willingness to participate (e.g. significant new information regarding the safety profile of the MRV-MNP, etc.) written informed consent will be repeated using the processes outlined below using an updated ethically approved ICD or ICD addendum as appropriate.

#### Overview of informed consent procedures

Whether undertaken in English or a local language, the ICD will be reviewed line-by-line with the participant/parent by the person undertaking the consent process ensuring all details are covered and understood. Self-reading alone is not considered to be sufficient. During this review, chances to ask questions and seek clarification will be given by the person taking consent. After this review, all participants/parents will be seen by a study clinician who will give them a further chance to ask questions and will also clarify any questions, particularly of a medical nature, that the person taking consent was unable to address. In addition, prior to signing/thumb-printing the ICD, the participant/parent must successfully complete an ‘Assessment of Understanding’ undertaken by the study clinician in the presence of a different member of the trial team to the individual undertaking the rest of the informed consent process. This series of questions, approved by the appropriate ethics committee and related to the content of the ICD, aims to confirm key information regarding the trial has been understood. The outcome of the assessment (enrol/repeat consent process/do not enrol) will be handled based on the study-specific procedure covering informed consent for the trial.

#### Consent by participants/parents who are English literate

When the participant/parent providing informed consent is literate in English, an impartial witness must not be present during the informed consent discussions and signature process.

English literacy will be confirmed by the individual undertaking consent. The potential participant/parent will be asked to read one or two paragraphs of the ICD and should then be asked to explain the meaning in their own words. If English literacy is confirmed, the entire informed consent process must take place in English. No local language should be used, e.g. to explain further difficult concepts. Therefore, if there is any doubt regarding the person’s level of English literacy, consent should take place in the most appropriate local language (this does not preclude the use of English but ensures that an impartial witness is present who will attest to the accuracy and completeness of the information provided).

An English-literate participant/parent will sign the ICD for themselves.

#### Consent by participants/parents who are not English literate

As the local languages in The Gambia are spoken and not widely written, the ICD is provided in written form in English only and translated directly into relevant local languages by the person undertaking consent. Previous attempts to translate and back-translate ICD into local languages have resulted in loss of meaning and thus are not used. A recording of the ICD in local languages is made prior to the initiation of consent procedures to ensure language use is consistent, accurate and agreed upon by all those taking consent. The accuracy of the agreed translation is confirmed by an individual external to the trial team, and this is documented.

When a participant/parent is not sufficiently literate in English, an impartial witness must be present throughout the informed consent discussions, assessment of understanding and signature process. Both the impartial witness and the individual taking consent must be fluent in English and in the local language used and this fluency must be documented in their curriculum vitae. The language in which informed consent is undertaken should generally be the language in which the individual being consented is most fluent (which may not be the tribe to which they belong). Impartial witnesses are not part of the clinical trial team but instead are recommended by the local community (for example, by the Alkalo [village head]) and attend the trial site on a rotational basis to ensure they remain independent.

A non-English literate participant may thumb-print or sign the ICD according to their preference. The impartial witness will time and date the signature/thumbprint on behalf of the participant.

At the end of the process, the impartial witness will also sign. In signing, they are attesting to the fact that all the information in the ICD has been given accurately and appears to have been understood by the person providing consent, that an opportunity has been given to ask questions and that these questions have been answered to the apparent satisfaction of the person providing consent and that consent is being given freely. This is the only role of the impartial witness. They should not in any way influence the decision-making of the participant/parent.

#### Consent by guardians

Consent can only be provided by a guardian rather than a biological parent if both biological parents have passed away or both parents are out of the country for a prolonged period and another adult is therefore bringing up the child. Guardians cannot consent if the parent is in another part of The Gambia or is out of the country only short term. As guardianship is very rarely transferred legally in The Gambia, anybody meeting the described criteria must sign a guardianship statement at the first visit, before informed consent procedures are undertaken. Biological parenthood will be established based on verbal report and will be documented. An additional source for this information is not required. The only exception to the above would be in the circumstances in which guardianship had been legally transferred to another adult and an official document was available to confirm this. A certified copy of such a legal document would be made under these circumstances. ‘Parent’ is used throughout the protocol but includes guardian under the conditions described.

#### Additional consent provisions for collection and use of participant data and biological specimens {26b}

As part of the process, informed consent will be sought for the future use of any remaining samples and associated data for ethically approved research which is expected to benefit the people of The Gambia. Such future use could include genetic testing. Failure to provide such informed consent will not preclude enrolment in the trial itself.

## Interventions

### Explanation for the choice of comparators {6b}

The trial will compare the administration of the MRV by MNP to the administration of the same vaccine by SC injection. In both cases, the MRV is manufactured by the Serum Institute of India Pvt. Ltd. The vaccine is licensed for SC injection under the Central Drugs Standard Control Organization of India and pre-qualified by the WHO for supply through UNICEF and other UN agencies. The vaccine meets the requirements of the WHO when tested by the methods outlined in WHO TRS 840 (1994).

To minimize the risk of bias in the safety or other data collected, the trial is of a double-dummy design meaning that all participants receive both an MNP and a SC injection. Participants who receive the MRV via the MNP receive a SC placebo injection (0.9% (weight/volume) sodium chloride). Participants who receive the MRV via SC injection receive a PLA-MNP. Neither the participants, nor the trial staff administering the MNP and SC injections, nor any staff involved in safety or other data collection will therefore be aware of the group to which an individual belongs. Given the importance of ensuring the safety data are robust, as this is the first use of the MRV-MNP in humans, this approach was felt to be warranted. As the MNP application is expected to cause little or no discomfort, the approach was also judged to be warranted in toddlers and infants who will therefore only get the one SC injection as per the normal EPI schedule.

### Intervention description {11a}

Both the SC injection (0.5 mL) and the MNP contain not less than 1000 CCID50 of the attenuated Edmonston-Zagreb measles and Wistar RA 27/3 rubella viruses.

The MNP are manufactured by Micron Biomedical, Inc. The MRV is incorporated into microneedles that dissolve and deliver the vaccine into the skin on application. The excipients from which the microneedles are made are of pharmaceutical-grade and are found on the United States (US) Food and Drug Administration (FDA)’s Inactive Ingredient Database for Approved Drug Products. The placebo microneedles contain the same excipients but do not include the measles and rubella virus antigens. The MRV-MNP and the PLA-MNP are visually indistinguishable in appearance and will be applied to the dorsal aspect of the wrist.

The MRV for SC injection is lyophilized and reconstituted with the sterile water diluent immediately before administration. The 0.9% (weight/volume) sodium chloride is presented in a sterile ampoule. The SC injections will generally be administered to the lateral aspect of the deltoid area in adults and to the lateral aspect of the thigh in toddlers and infants. All SC injections will be administered with a 0.5-mL fixed-dose, auto-disable syringe and 25-mm 23G needle.

The MNP will be applied for 5 min and removed before the SC injection is administered.

### Criteria for discontinuing or modifying allocated interventions {11b}

All participants receive a single MNP and a single SC injection at visit 1. If a participant was to have a severe, early allergic/anaphylactic reaction to the MNP (applied first), the SC injection would not be administered. In addition, a participant/parent can withdraw at any time and thus could withdraw having received the MNP and before the SC injection.

### Strategies to improve adherence to interventions {11c}

Both the MNP and SC injection will be administered at a single timepoint by study staff therefore adherence monitoring is not relevant. All participants will be monitored by staff while the MNP is in place to ensure it is not disrupted.

### Relevant concomitant care permitted or prohibited during the trial {11d}

Participants should not receive non-study vaccines during the trial, apart from any non-MRV administered as part of catch-up/national campaigns by the Ministry of Health. If an MRV SIA (generally targeting under 5-year-old children) occurs during the trial, the parents of participants will be asked not to have their child vaccinated during the campaign. All toddlers and infants will receive an additional SC dose of an MRV at the end of their participation in the trial. Other EPI vaccines due at 9 months in infants (yellow fever and bivalent oral poliovirus vaccine (bOPV) and at 15 to 18 months in toddlers (diphtheria, tetanus and pertussis and bOPV) will be administered at the day 42 post-vaccination visit.

Participants expecting to require immunosuppressant medication, intravenous immunoglobulins or blood products during their participation in the trial will be ineligible to be enrolled. However, no medication or treatment that is subsequently clinically indicated during the trial will be forbidden. Under such circumstances, safety follow-up will continue as planned to the degree possible. The decision to include the immunogenicity data for any such participants in the final analysis will be agreed between the sponsor and investigator prior to unblinding. Topical glucocorticoids for skin lightening and traditional and local herbal medications, including those applied topically, are not permitted during the trial.

### Provisions for post-trial care {30}

For health complaints which are judged to be unrelated to vaccination or trial participation — including conditions identified at screening, participants will receive initial care according to current established good medical practice within The Gambia. According to the nature of the complaint, such care (including, as appropriate, any additional investigations or treatment required) may be provided at the clinical trial site by study clinicians, within the MRCG CSD, or within government, non-governmental agency or private health care facilities. Decisions regarding the most appropriate location for treatment will be made on a case-by-case basis, involving the principal investigator and other senior clinicians in The Gambia when necessary. Such decisions will aim to ensure the care of the participant is in line with good medical practice based on current availability in The Gambia. For unrelated complaints, the trial team aim to ensure acute conditions are investigated and treated appropriately and that care for any chronic conditions is established. Long-term care for chronic conditions will not be provided by the trial team.

Any health complaints judged to be related to vaccination or trial participation will be investigated and managed in the most appropriate health care facility which may include health care facilities outside The Gambia. Financial cover to ensure such care, including for emergency evacuation, will be provided through the sponsor’s clinical trial insurance.

### Outcomes {12}

#### Study outcome measures

#### Primary — safety and tolerability

- The number, severity and relatedness of solicited local and systemic AEs collected on the day of study product administration and daily until day 13 following study product administration
- The number, severity and relatedness of unsolicited AE and SAE from the day of study product administration until day 180 following study product administration
- The number, severity and relatedness of biochemical and haematological abnormalities occurring until day 14 (adult cohort only) or day 7 (toddler and infant cohorts) following study product administration

#### Secondary — immunogenicity

#### Measles

- Measles SNA titres by plaque reduction neutralization test (PRNT)
- Measles serum IgG binding antibody concentrations by a bead-based multiplex assay

#### Rubella

- Rubella SNA titres by indirect immunocolorimetric assay (ICA)
- Rubella serum IgG binding antibody concentrations by bead-based multiplex assay

#### Adults and toddlers

- Geometric mean fold rise (GMFR) in measles and rubella SNA titres and IgG concentrations from baseline to day 42 following study product administration
- Percentage of participants undergoing seroconversion or experiencing a four-fold rise in measles and rubella SNA titres and measles and rubella IgG concentration between baseline and day 42 following study product administration
- Percentage of measles and rubella seroprotected participants based on SNA titres and IgG concentrations on day 42 and day 180 following study product administration
- Measles and rubella SNA GMT and measles and rubella IgG geometric mean concentrations (GMC) day 42 and day 180 following study product administration

#### Infants

- Percentage of participants undergoing seroconversion or experiencing a four-fold rise in measles and rubella SNA titres and measles and rubella IgG concentration between baseline and day 42 following study product administration
- Percentage of measles and rubella seroprotected participants based on SNA titres and IgG concentrations on day 42 and day 180 following study product administration
- Measles and rubella SNA GMT and measles and rubella IgG GMC day 42 and day 180 following study product administration

#### Exploratory

- To enumerate and characterize the measles and rubella-specific T-cell responses generated by MRV-MNP and MRV-SC
- To describe the experience of participants and parents following study product administration by MNP compared to SC injection
- To describe the views of trial personnel regarding study product administration by MNP and to SC injection

### Participant timeline {13}

The participant timeline is shown in Table 1.

**Table 1:** Participant timeline

Visit #	V0	V1	Home visits	V2	V3	V4	V5
Study day	−14 to −1	0	1 to 13	7	14	42	180
Visit window (days)	-	-	-	+2	+3	+14	+28
Written informed consent	X						
History and physical examination	X	X		X	X	X	X
Vital signs and anthropometry	X	X		X	X	X	X
Screening and safety labs	X			X	X^a^		
Serum pregnancy test^b^	X						
Urinary pregnancy test^b^		X					X
Malaria rapid diagnostic test/blood film	X	X					
Immunogenicity blood samples	X					X	X
Final confirmation of eligibility		X					
Randomization and study product administration		X					
Solicited local and systemic AE data collection		X	X	X	X		
Unsolicited AE collection		X	X	X	X	X	X
EPI vaccine administration^c^						X	
T-cell responses — exploratory	X			X		X	X
Participant/parent experience — exploratory		X X^d^			X	X	
End of study							X‖

^a^Adult cohort only; ^b^adult females only; ^c^toddlers and infants will be administered the other EPI vaccines due at 15 to 18 months and 9 to 10 months/12 months respectively at V4 (day 42);^d^pre-vaccination (may also be conducted at V0) and post-vaccination; ‖toddlers and infants will be administered an additional SC dose of a measles and rubella vaccine on or after the end of study visit outside the current protocol to ensure protection based on the current EPI schedule in The Gambia

### Sample size {14}

The sample size has not been determined by a power calculation based on testing a formal statistical hypothesis. Instead, it has been chosen to provide the required descriptive data on the safety and tolerability of the MRV-MNP to guide decisions regarding product development and to provide supporting information regarding the immunogenicity of the MRV-MNP.

#### Safety and tolerability

Table 2 indicates the probability that a given safety event will occur at least once or at least twice based on true event rates in the vaccinated population of between 1 and 20% based on the different sample sizes (*n*).

**Table 2:** The probability of any given safety event occurring at least once or at least twice given a true population event rate of between 1.0 and 20.0% according to the indicated samples sizes per group

True event rate (%)	*n* = 30		*n* = 60		*n* = 150	
	Probability of at least 1 event	Probability of at least 2 events	Probability of at least 1 event	Probability of at least 2 events	Probability of at least 1 event	Probability of at least 2 events
20	99.9	98.9	100.0	100.0	100.0	100.0
10	95.8	81.6	99.8	98.6	100.0	100.0
7.5	90.4	66.9	99.1	94.5	100.0	100.0
5	78.5	44.6	95.4	80.8	100.0	99.6
2.5	53.2	17.2	78.1	44.4	97.8	89.1
1	26.0	3.6	45.3	12.1	77.9	44.3

A sample size of 30 (adult) participants provides a probability of 95.8% that at least one episode of a given safety event will occur and a probability of 81.6% that at least two episodes of an event will occur based on a true event rate of 10% in the vaccinated population. The same sample size provides probability of 78.5%, 53.2% and 26.0% that at least one event will occur given a true event rate of 5%, 2.5% and 1% in the population. If a given safety event does not occur in 30 adult participants, we can be 95% confident that the true event rate in the population is less than 11.4%.

A sample size of 60 (toddler or infant) participants provides a probability of 99.8% that at least one episode of a given safety event will occur and a probability of 98.6% that at least two episodes of an event will occur in each of the given cohorts based on a true event rate of 10% in the vaccinated cohort. The same sample size provides probability of 95.4%, 78.1% and 45.3% that at least one event will occur given a true event rate of 5%, 2.5% and 1% in the cohort. If a given safety event does not occur in a cohort, we can be 95% confident that the true event rate in that cohort is less than 6.0%.

A sample size of 150 participants receiving the MRV-MNP overall provides a probability of close to 100.0% that at least two episodes of an event will occur in the whole study population based on a true event rate of 10% in the vaccinated cohort. The same sample size provides probability of close to 100.0%, 97.8% and 79.9% that at least one event will occur given a true event rate of 5%, 2.5% and 1% in the cohort. If a given safety event does not occur in a study, we can be 95% confident that the true event rate in that cohort is less than 2.5%.

Table 3 indicates the expected precision surrounding the estimates of given safety event rates in each cohort and in all participants who will receive MRV-MNP during the study. For example, if a fever (or any other event) is recorded in 5% of all participants who receive the MRV-MNP, we will be 95% confident that the true frequency of fever related to MRV-MNP administration lies between 2.5 and 9.8%.

**Table 3:** Expected precision around the given safety event rate estimates in adults, toddler/infants and the whole population who will receive MRV-MNP

Event rate in sample (%)	***n*** = 30	***n*** = 60	***n*** = 150
	Event rate (%) (95% CI)	Event rate (95% CI)	Event rate (95% CI)
20.0	20.0 (9.5–37.3)	20 (11.8–31.8)	20.0 (14.4–27.1)
10.0	10.0 (3.5–25.6)	10 (4.7–20.2)	10.0 (6.2–15.8)
7.5	7.5 (22–22.4)	7.5 (3.1–17.0)	7.5 (4.3–12.9)
5.0	5.0 (1.2–19.1)	5.0 (1.7–13.7)	5.0 (2.5–9.8)
2.5	2.5 (0.3–15.4)	2.5 (0.6–10.1)	2.5 (1.0–6.4)
1.0	1.0 (0.0–13.1)	1.0 (0.1–7.8)	1.0 (0.2–4.2)

#### Immunogenicity

Table 4 indicates the expected precision surrounding the estimates of given immune response rates in each cohort. For example, if the immune response rate in adults vaccinated with MRV-MNP is 60%, we will be 95% confident that the true immune response rate in adults lies between 42.3 and 75.4%. Similarly, if the immune response rate in infants is 80%, we will be 95% confident that the true immune response rate in infants lies between 68.2 and 88.2%.

**Table 4:** Expected precision around the given immune response (seroconversion or fourfold titre/concentration rise) rate estimates in adults and toddler/infants who will receive MRV-MNP

Immune response rate in sample (%)	***n*** = 30	***n*** = 60
	Immune response (%) (95% CI)	Immune response (%) (95% CI)
20.0	20.0 (9.5–37.3)	20.0 (11.8–31.8)
40.0	40.0 (24.6–57.7)	40.0 (28.6–52.6)
60.0	60.0 (42.3–75.4)	60.0 (47.4–71.4)
80.0	80.0 (62.7– 90.5)	80.0 (68.2–88.2)
100.0	100.0 (88.7–100.0)	100.0 (94.0–100.0)

### Recruitment {15}

#### Sensitization

Effective community and individual sensitization prior to trial initiation are key to trial recruitment and retention.

#### Community sensitization

Prior to any other activities taking place, a series of ‘kolanut’^1^ meetings with key leaders within the local community including the Alkalo (village/community leader), other community elders, religious leaders, members of women’s and mother’s groups and other community advocates will be undertaken at venues within the local area. During these meetings, details regarding the trial will be discussed and a chance given for questions to be asked to ensure everybody present gains a full understanding of the purpose of the trial and other key information (e.g. risks and benefits). The information provided will be based on the ICD to ensure the accuracy and consistency of the information given.

Following these meetings, information regarding the trial will be disseminated within the local community through well-established community networks. The meetings also serve a critical role in ensuring influential members of the community properly understand the trial and therefore that accurate information is passed on and misunderstanding is avoided. Feedback received during these meetings also assists the trial team in planning individual sensitization activities.

To recruit females using effective birth control, information will also be provided to staff of local family planning clinics so they can refer anyone who may be interested to the trial team. Toddlers and infants will predominantly be recruited through the government EPI clinics; therefore, the officers in charge of local health facilities as well as the public health officers who coordinate the clinics will be sensitized in the same way.

#### Individual sensitization

Individual sensitization involves the direct provision of more detailed information regarding the trial to those expressing potential interest in participation. During individual sensitization, members of the clinical trial team will provide a summary of the information in the approved ICD. At the end of this process, those who continue to express an interest in participating/having their children participate will be provided with the ICD and encouraged to discuss the trial with their partner, parent(s) and any other family members. Basic contact information including telephone numbers and a description of their place of residence will also be collected at this point to allow subsequent follow-up. In the case of the toddler and infant cohorts, the trial team will always speak directly by telephone or face-to-face with the second parent (unless there is no partner/second parent involved in the child’s care) and confirm they are also supportive of trial participation before formal informed consent takes place.

## Assignment of interventions: allocation

### Sequence generation {16a}

Randomization will be based on a pre-established computer-generated permuted block randomization scheme. Randomization will be stratified by sex although no fixed proportions of males and females will be required in any cohort. The block size will not be known to anyone involved in trial conduct.

### Concealment mechanism {16b}

Randomization will be undertaken using an in-house web-based electronic central randomization system to which only those unblinded staff undertaking randomization will have access. The system includes an audit trail which indicates who undertook randomization. The allocation sequence is concealed and the group to which a participant has been randomized only indicated after their subject number and eligibility for randomization has been confirmed.

### Implementation {16c}

The randomization scheme will be generated by a statistician not otherwise involved in the trial. Following screening and final confirmation of eligibility by a trial clinician, a unblinded member of the clinical trial team will undertake randomization. Having completed randomization, the same unblinded site personnel will prepare the appropriate MNP (MRV or placebo) and corresponding SC injection for administration.

The MRV-MNP and PLA-MNP are indistinguishable in appearance; thus, only the label of the primary packaging will be covered with an opaque label in this case.

The MRV and 0.9% sodium chloride for SC injection will be drawn into identical syringes for administration. However, given they may differ in colour slightly, once the injection has been drawn into the syringe and is ready for administration, the barrel of the syringe will be wrapped with opaque tape, masking the fluid, and ensuring the syringes are indistinguishable in appearance.

The MNP in its primary packaging and the syringe containing the SC injection will then be handed in a blinded fashion to blinded study personnel who will administer the vaccine.

In this way, the participants/parents as well as the staff administering the vaccine and all staff collecting endpoint data will be blinded to the study products administered.

The unblinded trial staff, who will also be responsible for all vaccine handling, storage and accountability procedures, will be restricted in number to ensure the integrity of the blind at the site. They will have no role in endpoint assessment.

All randomization and vaccine accountability procedures, including all documentation related to these processes, will be maintained in an area under separate access control. Access will be limited strictly to the unblinded team.

## Assignment of interventions: blinding

### Who will be blinded {17a}

The use of the double-dummy design, in which all participants receive an identical MNP and SC injection, ensures that the personnel applying the MNP and administering the SC injection, the participants/parents of participants, all staff involved in safety and other data collection, laboratory staff and staff involved in data management and analysis will be blinded to treatment allocation.

### Procedure for unblinding if needed {17b}

In the event of a medical emergency, the principal investigator (or designee) may require that the blind be broken for the participant experiencing the emergency when knowledge of the participant’s treatment assignment may influence the participant’s clinical care. The electronic central randomization system allows for unblinding under these circumstances under separate access control provide to the principal investigator.

If unblinding is required, every effort will be made not to unblind the participant/parent unless it is considered necessary for their welfare. In addition, the number of investigator staff who are unblinded will be minimized under such circumstances.

## Data collection and management

### Plans for assessment and collection of outcomes {18a}

#### Safety

#### Definitions

##### Adverse event

Based on ICH-GCP E6 (R2), an AE in this trial is defined as any untoward medical occurrence in a participant administered a study product which does not necessarily have a causal relationship with the study product itself. An adverse event can therefore be any unfavorable and unintended sign (including an abnormal laboratory finding), symptom or disease temporally associated with the administration of the study product whether related to the study product or not. Symptoms, signs or conditions present at screening (visit 0) which do not change are not AE and will be recorded as part of the screening procedures. Any subsequent change in the severity of a symptom, sign or condition following screening will be recorded as AE.

##### Serious adverse event

An AE is defined as serious if it:

- Results in death
  Any deaths will be reported as grade 5 severity
- Is life threatening
  The term life-threatening in the definition of serious refers to an event in which the participant was at risk of death at the time of the event; it does not refer to an event which hypothetically might have caused death if it was more severe.
- Requires inpatient hospitalization or prolongation of existing hospitalization
- Results in persistent or significant disability/incapacity
- Is a congenital anomaly/birth defect

##### Important medical event

Important medical events that may not be immediately life threatening or result in death or hospitalization, but which may jeopardize the participant or may require intervention to prevent one of the other outcomes listed in the definition of serious should be reported in the same ways as serious adverse events.

##### Suspected unexpected serious adverse reaction

AE that are serious or are important medical events, that are judged to be related to study product administration, and that are unexpected based on the information contained in the reference safety information will be termed suspected unexpected serious adverse reactions (SUSARs)

#### Solicited adverse events

The following solicited local and systemic adverse events will be collected and graded for severity based on the National Institute of Health (NIH), Division of AIDS (DAIDS) Table for Grading the Severity of Adult and Pediatric adverse events (version 2.1. Jul 2017):

- Adult local: pain, redness/erythema, swelling/induration, pruritis
- Toddler and infant local: pain, redness/erythema, swelling/induration
- Adult systemic: acute allergic reactions (day 0 only), axillary temperature, vomiting, diarrhoea, headache, fatigue, myalgia, arthralgia and rash
- Toddler and infant systemic: acute allergic reactions (day 0 only), axillary temperature, vomiting, diarrhoea, irritability, drowsiness, appetite and rash

Solicited AE will be recorded on the day of study product administration (visit 1 — day 0) by the study clinician and daily through home visits conducted by trained field workers between day 1 and day 13 post-study product administration. Any local and systemic AE ongoing after day 13 will be recorded by the study clinician as an unsolicited AE at the day 14 clinic visit (visit 3). Any grade 3 solicited AE identified during home visits will prompt an unscheduled clinic visit and review by a clinical study clinician on the same day or within 24 h at the latest.

Solicited AE data from home visits will be reviewed daily by a study clinician. In addition, home visits will be spot checked by senior members of the field team to ensure the quality and consistency of findings.

#### Unsolicited adverse events

Any event fulfilling the definition of an AE, but which is not reported based on the definition of solicited local and systemic AE will be reported as unsolicited AE. When possible, collections of individual signs and symptoms will be reported as the underlying clinical syndrome. For example, gastroenteritis should be reported rather than diarrhoea, vomiting and fever. If an underlying clinical syndrome is not apparent, symptoms and signs will be reported individually. Unsolicited AE will be coded by preferred term (PT) and primary system, order, class (SOC) for reporting according to the latest online version of the MedDRA®.

#### Classification of adverse events

##### Severity

Solicited local and systemic AE as well as unsolicited AE will be graded for severity based on the NIH DAIDS Table for Grading the Severity of Adult and Pediatric AE (version 2.1. Jul 2017) or, if not included, based on the criteria set out in Table 5. The highest severity grade applicable at any point during an illness will ultimately be reported. Any AE which results in death will be defined as severity grade 5.

**Table 5:** Severity grading for unsolicited adverse events^a^

		Description
Grade 1	Mild	No interference with activity and no or minimal intervention^1^ required
Grade 2	Moderate	Some interference with activity or requires more than minimal intervention
Grade 3	Severe	Prevents daily activity and required significant medical intervention
Grade 4	Life-threatening	Life-threatening consequences requiring urgent medical intervention
Grade 5	Death	Results in death

^a^Adapted from Cancer Therapy Evaluation Program, Common Terminology Criteria for AEs, version 3.0, DCTD, NCI, NIH, DHHS March 31, 2003, published on August 9, 2006

^1^One or two doses of antipyretic or simple analgesic medication or local topical treatment

##### Causality

Other than solicited local reactions which, by definition, are related to study product administration, other AEs will be assessed for relatedness to the study vaccine by a study clinician.

The relatedness of a particular AE will be assessed based on clinical judgment considering the timing of the event in relation to study product administration, the nature of the event, the presence or absence of other illnesses or conditions to explain the event and relevant background history and concomitant medication use.

Based on these assessments, the relationship between a given AE and study product will be defined as:

- Related^2^: There is a reasonable possibility of a causal relationship between the AE and the study product administered. The AE is more likely to be explained by the administration of the study product than by another cause.
- Not related: There is not a reasonable possibility of a causal relationship between the AE and the study product administered. The AE is more likely to be explained by another cause.

Given the double-dummy design, at the time of the initial unblinded assessment of an AE, it will not be possible to establish whether systemic AE is related to the MRV-MNP or MRV-SC.

##### Expectedness

Expectedness, either ‘expected’ or ‘unexpected’, will be assessed for unsolicited related AE by the sponsor’s medical expert based on the latest Investigator’s Brochure for the MRV-MNP and summary of product characteristics (SmPC) for the SC injection. For systemic events, the SmPC will serve as the reference safety information for the purposes of assessing expectedness of any reactions to the MRV irrespective of administration methods. Therefore, any systemic reaction judged to be expected based on the SmPC for the MRV will also be defined as expected following MRV-MNP.

**Outcome**

The outcome of AE will be defined as resolved/recovered, resolved/recovered with sequelae, ongoing stable chronic condition, ongoing at end of the study visit, resulted in death and unknown.

##### Safety laboratory investigations

Screening (visit 0) and safety (visit 2 and visit 3 [adults only]) laboratory investigations will be performed in the MRCG CSD laboratories according to their Standard Operating Procedures (SOPs). The SOPs govern the processes of sample reception, sample processing and result reporting. The CSD biochemistry, haematology, microbiology and serology laboratories are all ISO15189 accredited and Good Clinical Laboratory Practice (GCLP) compliant.

All abnormal safety labs will be graded based on a locally appropriate grading scale and will be judged for clinical significance and relatedness. Abnormal safety labs will be repeated as clinically indicated.

##### Immunogenicity

Serum samples for measles and rubella serological testing will be shipped to the Centers for Disease Control and Prevention, National Center for Immunization and Respiratory Diseases, Division of Viral Diseases) for processing according to CDC SOP and Quality Control (QC)/Quality Assurance (QA) procedures.

Measles-specific SNA titres will be measured using the PRNT. A standardized PRNT protocol as recommended by the WHO will be used as previously reported [67, 68].

Rubella-specific SNA titres will be measured using the ICA as previously reported [69].

Measles and rubella-specific IgG concentrations will be measured using a multiplex bead array (MBA) assay established at the CDC [70].

Both the measles and rubella SNA titres and the measles and rubella IgG concentrations will be calibrated to the appropriate WHO standards to facilitate comparisons. The reference standard for measles will be WHO/BS/06.2031 (WHO International Standard, 3rd International Standard for Anti-Measles) and for rubella will be RUBI-1-94 (WHO International Standard; Anti Rubella Immunoglobulin, Human) [71, 72].

Measles seropositivity will be defined as a standardized titre of > 120mIU/mL.

Rubella seropositivity will be defined as a standardized titre of > 10IU/mL.

### Plans to promote participant retention and complete follow-up {18b}

During sensitization and informed consent procedures, the commitment that the trial involves will be emphasized to ensure participants/parents of participants do not join the trial unless they feel that they will be able to attend for the required visits and make time for the required home visits. Blood sample bottles containing a local juice (Wonjo) which has the appearance of blood are used to illustrate the blood volumes required given that this can be sensitive. At all planned visits, the participants/parents of participants will be reminded of the need to contact the study team both if they have any concerns about their/their child’s well-being and if they have any unexpected travel plans.

In addition, to facilitate communication, a telephone on a CUG network with all members of the field team will be provided to participants/parents to ensure they are able to contact the investigator team at any time day or night without the need for telephone credit.

Irrespective of the reason for withdrawal or discontinuation of a participant, the investigator should make every reasonable effort to ensure the safety of the participant. This includes — in order of general priority, continuing to:

- Assess and provide the clinical care for AE that would have occurred had the participant remained in the study or to ensure such assessment and care is available to the participant
- Undertake the safety follow-up planned in the study including the planned safety bloods
- Capture planned safety data in the clinical trial database

### Data management {19}

All data management activities will be described in the Data Management Plan for the trial.

#### Data collection

Data collection in the field will be undertaken by members of the investigator team who are responsible for ensuring its accuracy, completeness, legibility and timeliness. Data required to assess non-laboratory-based trial endpoints will be collected onto electronic case report forms (eCRF). A source document designation log will define the source for all information to be collected. Some information will be collected directly into an eCRF based on the participant report; thus, the eCRF will be the source in this case. Additional source documents include other trial-specific documents (vital signs and anthropometry cards; sample collection logs; study product administration logs; study product accountability and cold-chain documentation; randomization documents; screening and enrolment log; trial clinical progress notes etc.) as well as non-trial-related documents (clinical records from any hospital/clinic admissions, printouts of clinical laboratory results and prescription charts, etc.).

Serological data from external laboratories will be received by MRCG electronically in a secure format and integrated with data in the clinical trial database for analysis. An original set of all external laboratory data received will be retained for future monitoring and audit purposes.

#### Clinical trial database

Trial data will be collected through eCRF into a clinical data management system (CDMS) designed within a 21 CFR Part 11 compliant with the REDCap™ platform. The platform allows data to be captured online but also offline. This is important given internet connectivity may be unreliable, particularly when data are being collected in the field through home visits.

The trial database will be developed by the database developer in liaison with the trial data manager as per the MRCG data management SOP on Database Development. Database validation will be documented prior to release

#### Data validation and cleaning

Data validation will be undertaken to identify missing, erroneous, implausible and inconsistent data and to identify protocol deviations — for example related to participant eligibility or visit windows. A combination of on-entry and batch validation will be used. All validations and procedures involved in data cleaning, performed manually or automatically by validation check programming, will be defined in the data management plan. Data queries will be raised real time based on the defined error checks for review and resolution by the investigator team through a defined change process maintaining a clear audit trail.

#### Data coding

All AE data will be coded by PT and primary SOC according to the latest online version of MedDRA®. Generic rather than brand names will be used for all concomitant medication.

#### Data security and storage

All data will be stored on access-controlled computers and servers in line with applicable MRCG data management and data security policies. Databases will be backed-up as part of MRCG information technology disaster recovery policy.

### Confidentiality {27}

All clinical trial data and samples will be collected and stored in a linked-anonymized format using the participant’s screening number. No participant identifiable information, or collection of data which would otherwise allow a participant to be identified by those outside the investigator team, will be stored in either the clinical trial database or with the trial samples.

Participant identifiable information (name, address, contact telephone numbers etc.) and information linking the screening and randomization numbers to the participant identifiable information will only be available to the investigator team collecting and storing the data. Such information will be stored in locked filing cabinets or other lockable storage devices at the clinical trial sites and will only be accessible to those members of the investigator team requiring such information for day-to-day trial conduct. The information will also be held in a secure, access restricted database which will be used to track participants and facilitate visit planning. This database is unrelated to the main clinical trial database and will only be accessible to those members of the investigator team requiring access for study coordination.

Clinical research associates and external auditors conducting on-site monitoring or audit would necessarily be given access to participant identifiable information to complete their work (e.g. to confirm the completeness of the participant identification code list or the accuracy of ICD completion). Information regarding who will have access to such data and confirming the purpose of such access will be included in the ICD. No participant identifiable data will be made available to other parties or laboratories involved in the trial or to other external researchers.

### Plans for collection, laboratory evaluation and storage of biological specimens for genetic or molecular analysis in this trial/future use {33}

No genetic or molecular analysis is planned as part of this study. However, informed consent will separately be obtained for the future use of samples, including for genetic analysis. Failure to provide such consent will not preclude participation in this trial.

## Statistical methods

### Statistical methods for primary and secondary outcomes {20a}

Full details of the statistical methods to be employed are found in the statistical analysis plan for the trial. All analysis will be descriptive. Baseline demographic and anthropometric data as well as the defined safety and immunogenicity outcome measures will be tabulated by cohort and group with appropriate measures of spread.

### Interim analyses {21b}

A report describing the safety data to day 14 post-study product administration for the adult and toddler cohorts will be prepared for review by the independent DMC by a statistician allocated to this role but not otherwise involved in the analysis of the trial. The data stratified by group will be available to the DMC members alone and will be reviewed in a closed session of the committee.

The following will prompt a pause to further recruitment and study product administration pending a formal unblinded safety review by the DMC:

- Any SUSAR
- Any death in a trial participant related to study product administration
- Any grade > 3 solicited local AE
- Any grade > 3 solicited systemic AE or grade > 3 clinical laboratory abnormality judged related to vaccination
- The occurrence of the same grade > 2 solicited systemic AE event or grade > 2 clinical laboratory abnormality judged to be related to vaccination in 20% or more of participants (based on the number of participants dosed at any given timepoint)

The trial could be terminated based on the occurrence of the above safety events or based on the planned day 14 review of the safety data for each cohort. If the DMC did not feel either further dosing within the cohort or age de-escalation respectively was supported by the accumulating safety data, their advice would be provided to the sponsor who would make the ultimate decision in discussion with the investigators and other external experts if judged warranted. However, it is unlikely the sponsor would not follow the advice of the DMC

No interim analysis of immunogenicity data will be performed.

### Methods for additional analyses (e.g. subgroup analyses) {20b}

No additional or subgroup analyses are planned.

### Methods in analysis to handle protocol non-adherence and any statistical methods to handle missing data {20c}

Final study data will be reviewed in a blinded fashion by the investigators and sponsor, and decisions regarding analysis populations made prior to unblinding. This review will focus on protocol deviations including, but not limited to, any violations of inclusion and exclusion criteria and out-of-window visits and samples. Based on this review a primary safety population and a primary immunogenicity population will be defined. The primary safety population will generally include all participants who had a study product administered and who subsequently provided any safety data. The primary immunogenicity population will exclude any participants with protocol deviations that are expected to significantly impact on the vaccine immunogenicity.

The reasons data are missing will be defined during the blinded data review and any impact on the safety and immunogenicity endpoints determined. It is expected that any missing data will be considered to be missing at random. Data will not be estimated or imputed.

### Plans to give access to the full protocol, participant-level data and statistical code {31c}

Data collected in the study including de-identified participant data, the data dictionary and additional documents including the study protocol and statistical analysis plan will be made available in line with the WHO Statement on the Public Disclosure of Trial Results.

## Oversight and monitoring

### Composition of the coordinating Centre and trial steering committee {5d}

All aspects of the trial will be coordinated through regular meetings including the sponsor, sponsor’s medical expert, principal investigator, clinical trial coordinator, data manager and project manager. Additional personnel, including staff from the CDC, responsible for all serological analysis, trial statisticians and additional representatives of the sponsor, investigator and other partners will be invited to these meetings as required according to the phase of the trial.

### Composition of the data monitoring committee, its role and reporting structure {21a}

The DMC for the trial will be constituted of four members, all of whom will be independent of both the sponsor and investigator. The DMC will include three members with combined expertise in adult and paediatric medicine, clinical vaccine trials, MNP technology and clinical research in LMIC in sub-Saharan Africa, as well as an independent DMC statistician.

The DMC will review all safety data, and data on protocol adherence, to day 14 following study product administration in the adult and subsequently the toddler cohort and provide advice to the sponsor on whether age de-escalation should continue as planned. The DMC will also meet if a pause rule is met during trial conduct and will recommend any additional measures to the sponsor judged to be required. Full details of the DMC’s function and workings are provided in the DMC charter.

### Adverse event reporting and harms {22}

See the “Plans for assessment and collection of outcomes {18a}” section.

### Frequency and plans for auditing trial conduct {23}

No audit of the trial is planned although the trial could be subjected to a regulatory audit by the Medicines Control Agency, the national regulatory agency in The Gambia.

### Plans for communicating important protocol amendments to relevant parties (e.g. trial participants, ethical committees) {25}

Any protocol amendments required during the conduct of the trial will be agreed between the sponsor and investigator. All amendments will be submitted for review and approval by the Government of The Gambia/MRC Joint Ethics Committee, the LSHTM Research Ethics Committee and the Medicines Control Agency. If the amendment is considered to have any significant impact on participants already enrolled in the trial, they will be informed of this. Under these circumstances, the informed consent process will be repeated using a revised ICD or appropriate ICD addendum.

## Dissemination plans {31a}

The results of the trial will be published in open-access, peer-reviewed medical journals and will be presented at relevant biomedical conferences as well as to other stakeholders. The results will be fed back to study participants/parents of study participants during Open Days held at the trial recruitment site to which participants/parents of participants will be invited.

## Discussion

The protocol describes a phase 1/2, single-centre, double-blind, double-dummy, randomized, controlled, age de-escalation, trial of a measles and rubella vaccine microneedle patch being conducted at MRC Unit The Gambia at LSHTM. The trial is sponsored by Micron Biomedical, Inc. Recruitment and follow-up in the trial are currently ongoing and proceeding as planned. The trial has not ultimately been significantly impacted by the COVID-19 pandemic given that appropriate safety measures for participants and staff, and strategies to mitigate any negative impact of the pandemic on study conduct, were established prior to trial initiation

## Trial status

Protocol version 3.1 dated 26 July 2021. The first participant was screened on 18 May 2021. The first participant was vaccinated on 24 May 2021. Recruitment is expected to be completed around June 2022 (last participant, first visit).

## Data Availability

The final dataset from the trial will be owned by the trial sponsor, Micron Biomedical, Inc, but will additionally be made available to the trial investigators.

## Abbreviations

AE: Adverse event
AFR: African region
AMR: Americas region
BMI: Body mass index
bOPV: Bivalent oral poliovirus vaccine
BP: Non-invasive blood pressure
CDC: Centers for Disease Control and Prevention
CDMS: Clinical Data Management System
CI: Confidence interval
CRS: Congenital rubella syndrome
CSD: Clinical services department
CUG: Closed user group
DAIDS: National Institute of Health, Division of AIDS
DMC: Data Monitoring Committee
°C: Degrees Centigrade
eCRF: Electronic case report form
EIA: Enzyme-linked immunoassay
EMR: Eastern Mediterranean region
EPI: Expanded Programme on Immunization
EUR: European region
FDA: Food and Drug Administration
IgG: Immunoglobulin G
GCLP: Good Clinical Laboratory Practice
GMC: Geometric mean concentration
GMFR: Geometric mean fold rise
GMT: Geometric mean titre
GVAP: Global Vaccine Action Plan
HAI: Haemagglutination inhibition
HIV: Human immunodeficiency virus
ICA: Indirect immunocolorimetric assay
ICD: Informed consent document
ICH-GCP: International Committee on Harmonization Good Clinical Practice
IIV: Inactivated influenza vaccine
IM: Intramuscular
IU/mL: International units per millilitre
IWC: Infant Welfare Card
kg/m^2^: Kilogramme per metre squared
LMICs: Low- and middle-income countries
MBA: Multiplex bead array
MCV: Measles containing vaccine
MedDRA: Medical Dictionary of Regulatory Affairs
mg/kg: Milligrammes per kilogramme
mIU/mL: Milli-International Units per millilitre
mL: Millilitre
mm: Millimetre
MNP: Microneedle patch
MOG: Myelin oligodendrocyte glycoprotein
MRCG: MRC Unit The Gambia at the London School of Hygiene and Tropical Medicine
MRV: Measles and rubella vaccine
MRV-MNP: Measles and rubella vaccine microneedle patch
MRV-SC: Measles and rubella vaccine by the subcutaneous route
N&S: Needle and syringe
PFU: Plaque forming units
PLA-MNP: Placebo microneedle patch
PLA-SC: Placebo subcutaneous injection
PRNT: Plaque reduction neutralization test
PT: Preferred term
QA: Quality assurance
QC: Quality control
RDT: Rapid diagnostic test for malaria
SAE: Serious adverse event
SAGE: Strategic Advisory Group of Experts on Immunization
SAR: Special Autonomous Region
SC: Subcutaneous
SD: Standard deviation
SEAR: Southeast Asia region
SIA: Supplementary immunization activities
SmPC: Summary of product characteristics
SNA: Serum neutralizing antibodies
SOC: System order class
SOP: Standard operating procedure
SSPE: Sub-acute sclerosing panencephalitis
SUSAR: Suspected unexpected serious adverse reactions
US: United States
WHA: World Health Assembly
WHO: World Health Organization
WPR: Western Pacific region

## Footnotes

1 Kola nuts (the seeds of *Cola nitida* and *Cola acuminata*) are given to members of the community at the end of the meeting as a sign of thanks and respect. Their acceptance by the community can be taken to indicate support for the trial

2 Definition based on the revised European Commission ‘Detailed guidance on the collection, verification and presentation of AE/reaction reports arising from clinical trials of medicinal products for human use’.
